# A randomized field experiment comparing nudges and boosts for household electricity conservation in student housing

**DOI:** 10.1038/s41598-026-69386-6

**Published:** 2026-09-03

**Authors:** Yavor Paunov, Till Grüne-Yanoff, Caterina Marchionni, Claudia Schwirplies

**Affiliations:** 1 Division of Philosophy and History of Technology, KTH Royal Institute of Technology, Teknikringen 76, 10044 Stockholm, Sweden; 2 School of Social Sciences, Department of Econonic and Consumer Psychology, University of Mannheim, A 5, 6 – Raum C 211, 68159 Mannheim, Germany; 3 Faculty of Social Sciences, University of Helsinki, PL 24 (Unioninkatu 40), 00014 Helsinki, Finland; 4 School of Business & Economics, Marburg University, Universitätsstraße 24, 35032 Marburg, Germany

**Keywords:** Environmental social sciences, Environmental studies, Psychology, Psychology

## Abstract

Behavioral interventions can promote household energy conservation, yet evidence on the mediators and moderators driving their effects remains limited. In a 24-week randomized field experiment with 140 student households, we contrasted two behavioral interventions: a social comparison “nudge” with a competence-enhancing “boost”. Specifically, the nudged group received weekly normative feedback on their electricity use relative to their peers, while the boosted participants accessed device-specific energy-saving tips. Both treatments significantly reduced electricity consumption relative to control by attenuating the seasonal increase observed among untreated households, corresponding to avoided consumption of ~7 MWh. Exploratory analyses indicated that the effects were concentrated among participants with higher baseline consumption. Within this group, the Boost intervention was associated with a statistically significant reduction of 3.97 kWh per week relative to control. The nudged participants reduced their weekly consumption by 3.12 kWh. While both interventions performed similarly over time, evidence for competence enhancement was limited: competence scores increased modestly in the boost group relative to control, but this effect became marginal after correcting for multiple testing. Further evidence indicates that intervention effects were strongest among participants with higher pre-treatment competence and environmental motivation, suggesting a moderating role of these variables.

**Supplementary Information:**

The online version contains supplementary material available at https://doi.org/10.1038/s41598-026-69386-6.

## Introduction

Behavioral interventions (BIs) are used to promote sustainable outcomes such as energy conservation. Reducing energy consumption is desirable both for individual households (by reducing utility costs) as well as for society at large. While most BIs share similar benevolent goals, the strategies they employ vary substantially: Nudges^1^ leverage cognitive biases and subtle environmental changes to induce energy savings, while boosts^2,3^ promote energy savings by enhancing people,s conservation competences^4^.

This distinction relies on specifying the mechanisms through which boosts and nudges influence behavior. A useful way to situate it is the COM-B model, which conceptualizes behavior as a function of capability, opportunity, and motivation^5^. From this perspective, boosts primarily target capability: they aim to increase people,s psychological capacity to act effectively by improving knowledge, skills, and competences. In the energy domain, a boost could improve residents, conservation capabilities by providing device-specific, actionable energy saving strategies^6^. Nudges, by contrast, typically alter the choice environment or make particular cues more salient, thereby shaping motivation and opportunity without necessarily expanding the person,s underlying capability.

A mechanistic distinction also clarifies why boosts often require active cooperation from the target individual^7^. A boost can only work if people engage with competence-enhancing information and incorporate it into their behavioral repertoire. This is consistent with self-determination theory, where perceived competence is understood as an important basis for self-directed motivation^8^. On the other hand, a whole class of normative feedback nudges rely on a different pathway: by making descriptive and injunctive norms salient, they can motivate behavior change through social comparison and perceived peer approval^9,10^. Thus, both nudges and boosts can promote energy conservation, but they do so through theoretically distinct mechanisms.

Distinguishing BIs by mechanism is also important for testing theoretical assumptions in the field^11^, for transferring effects across settings^12^, and for predicting the durability of intervention outcomes^13^. Once a causal variable has been identified in a given context, it can be measured in different target populations to predict intervention success there. For example, prior research links pre-treatment consumption to the effectiveness of nudging interventions such as gamification^14^ and normative feedback provision^15^ in the domain of energy saving. Accordingly, baseline consumption has been shown to be an influential predictor in other settings, such as water conservation^16^ and air conditioner use^17^.

Furthermore, accurate mechanistic identification is tightly linked to the longevity of intervention outcomes. For nudges, if automatic processes such as norm activation^18^ are repeatedly triggered to form beneficial consumption habits, then one can expect that intervention outcomes will remain stable over time. Likewise, interventions that build energy-saving competencies^4,6^ can equip people with lasting skills and confidence, enabling them to sustain energy saving behaviors without ongoing external support.

Despite the importance of mechanistic information for successful behavioral policy, surprisingly little empirical research tests mechanistic hypotheses in a direct comparison between nudges and boosts. This gap is further exacerbated by methodological shortcomings. A recent literature review^19^ showed that 80% of the mechanistic evidence linking household energy saving interventions to behavioral outcomes is correlational. To help fill this gap, we conducted a 24-week field experiment across 140 student housing units in Stockholm, Sweden, contrasting the effects of a classic nudge-type intervention^20^ with a boost-type treatment^6^ on household electricity consumption. Our specific objectives were to compare the cumulative effects of each intervention, to track energy savings over time, and to explore the mechanisms behind both intervention strategies.

We chose normative feedback as the nudge exemplar. In such interventions, participants receive regular feedback about their energy consumption along with information about the average consumption of their peers. An accompanying message serves as an indicator for peer approval or disapproval of consumption behavior^15,20^. Foundational research on normative behavior^18^ suggests that peer feedback increases norm salience, inducing people to adjust their behavior and meet the social norm.

For the boost, we opted to provide short actionable energy-saving strategies which can expand people,s domestic energy saving competence. We define such competence as the ability to successfully execute energy-saving behaviors at home. Key household devices, such as ovens, microwaves, and fridges, were labelled with QR code stickers, cf^6^. Scanning the QR codes provided users with weekly device-specific energy-saving tips. Examples included microwave cooking recipes, freezer defrosting procedures, and radiator cleaning instructions. Theoretical research^2^ suggests that offering contextually appropriate and actionable energy-saving strategies can enhance consumption competencies, thereby promoting sustainable behavior.

### Hypotheses

Our mechanistic considerations led to four preregistered hypotheses. Normative feedback was expected to reduce electricity consumption by increasing the salience of descriptive and injunctive social norms. The Boost intervention, however, directly supplied participants with concrete and actionable strategies for reducing energy use. We therefore expected the Boost intervention to produce a stronger overall behavioral effect than the Nudge intervention.

**H1.** Participants in the Boost condition will consume less electricity during the intervention period than participants in the Nudge condition.

As competence enhancement was the primary causal target of the Boost intervention, participants receiving the energy-saving tips were also expected to acquire greater domestic energy-saving competence than participants receiving normative feedback.

**H2.** Participants in the Boost condition will perform better on a final domestic energy-saving competence quiz than participants in the Nudge condition.

Next, as competence acquisition and the routinization of newly acquired energy-saving strategies were expected to develop gradually, we expected that the difference between the two interventions to increase over time, as participants in the Boost condition acquired and repeatedly applied the provided strategies.

**H3.** The difference in electricity consumption between the Boost and Nudge conditions will become more pronounced toward the middle and end of the intervention period.

Following the same rationale, we expected the effectiveness of the Boost intervention to increase progressively as participants expanded and applied their energy-saving competence.

**H4.** Participants in the Boost condition will progressively reduce their electricity consumption over the course of the intervention period.

A no-intervention Control group was used as a baseline, allowing treatment-versus-control comparisons to serve as benchmark estimates of whether each intervention changed consumption relative to no intervention. These benchmark comparisons were not preregistered.

In addition to domestic energy-saving competence, we explored other potential drivers of the intervention effects. Previous research suggests that enhancing people,s competences can increase their sense of self-efficacy and motivation ^21^. We also administered a norm salience measure to examine whether participants in the Nudge condition exhibited a larger pre-to-post difference in norm salience than participants in the Boost condition. A detailed description of the experimental design, procedure, and independent measures is available in the Methods section.

## Methods

## Ethics statement

This research was approved by the Swedish Ethical Review Authority. All methods were performed in accordance with relevant ethical guidelines and regulations. All respondents received equal remuneration for their completed participation in the experiment (a total of 30€ paid out in Amazon vouchers). The respondents provided written informed consent to participate after initial briefing and had the opportunity to stop their participation anytime and withdraw their data without providing a reason. All user related data has been pseudo - anonymized and were processed so that no unauthorized persons can access them. The data were processed in accordance with the General Data Protection Regulation (GDPR) and other applicable laws. All hosting services have been provided by EU-based servers. The participants could contact the chief experimenter and KTH,s data protection officer at any time to ask questions or request deletion of their data.

### Participant recruitment and sample characteristics

Two student housing providers in the Stockholm region of Sweden have been contacted for permission to recruit on their premises. Upon agreement, a team of four recruiters approached potential participants in person and provided flyers and a short instruction on how to register. A total of 159 participants were recruited and signed an informed consent to participate. Fifteen participants were excluded on the analysis level due to missing electricity consumption observations during their observation period (operationalized as 50% or more missing data points). This exclusion criterion was specified in our pre-registration. Two more participants requested to be excluded from the experiment by contacting the team, and the consumption data for two further users came in as duplicate. A total of 140 participants were included in the analysis (Control: n = 50; Nudge: n = 46; Boost: n = 44). Baseline characteristics per condition are reported in Supplementary Table S12.

We collected age, sex, education, income and household size data from 98 participants who completed the project,s welcome survey. Forty-two per-cent were female (n=41), 56% male (n=57), and one participant preferred not to share their biological sex. The largest proportion of the participants had a bachelor,s degree (43%, n=42), followed by respondents who obtained a master,s diploma (36%, n= 35). The majority of the participants (41%, n=40) earned less than 1000 € a month. The mean age of the respondents was 26 years (SD= 4.05), and the average household size was 1.5 (SD=.61).

### Experimental design and procedure

After scanning a QR code on a recruitment flyer, each participant was redirected to a custom website, which hosted the experiment. There, the participants were briefed and expressed their agreement to participate. After that, they were prompted to create password protected accounts. Each account was automatically associated with electricity consumption data on the backend. At this point, each participant was randomly allocated to one of 3 experimental conditions: a boost condition, a nudge condition, and a control. Random assignment produced groups that were statistically comparable on observed baseline characteristics, including pre-treatment electricity consumption, housing provider, age, household size, and baseline competence and motivation (Supplementary Table S12).

The interventions in each condition were administered on personalized dashboard pages. Each dashboard contained a short, animated video, which presented the instructions for the respective condition. The dashboard also contained a link to a welcome survey, where we captured the participants, scores on our competence, perceived self-efficacy, and norm salience measures. The measures were self-reported and were administered again in an exit survey at the end of the experimental period. For a full list of all scales, items, and instructions, please refer to “Measures.pdf” in the OSF directory (see Data Availability Statement for the link). Upon completing the Welcome Survey, the participants were immediately awarded for it (10€). The rest of the experimental reward (20€) was provided after completing the exit survey.

In the nudge condition the participants received graphical feedback for their weekly electricity consumption, compared with the consumption of the top 15% most efficient households in the project and the average consumption values of all other participants. The feedback was presented both graphically and in text. Injunctive feedback was provided by different smiley emojis, which varied depending on the respondent,s consumption. A happy green emoji signified approval in cases when the participant,s weekly consumption was lower than the lowest 15% bracket. A yellow smiley was presented when the respondent,s consumption was below the grand average, but more than the top savers, and a red “frown” emoji was presented when the respondent,s consumption exceeded the weekly use of both comparison categories. Supplementary Figure S5 presents a screenshot from a respondent,s dashboard in the nudge condition. To allow for continuous weekly comparisons, the dashboard also contained an interactive histogram depicting the historical electricity consumption values for the respective participant. A red line represented the average of all other participants in the project. The participants received weekly email reminders to check their weekly consumption when it was uploaded on their dashboards.

In the control treatment, the participants received no consumption feedback or social comparisons. Instead, they were only asked to complete the welcome and exit surveys.

In the boost treatment, consumption feedback was provided without social comparison elements (the respondents were exposed only to the weekly histogram update without references for the consumption of others). In addition, the participants received a set of additional experimental materials by post shortly after registering. The envelope contained an instruction letter, an A5 poster, and a set of four durable stickers. The poster listed common household appliances and color-coded them from most to power hungry (red for most, yellow for mid and green for least consuming). The stickers contained a label and a QR code. The label indicated where the sticker should be placed (close to stove/cooking area, sink, fridge, shower). The QR codes were associated with the participant account on the backend and were unique per appliance and housing unit. Each week, a new area-specific energy saving tip was uploaded to our platform and was made available by scanning the QR code with a phone. Scanned tips were automatically saved in the users, accounts and could be accessed every time when they log in their online accounts (under a tab, called “Energy saving tips”). On the backend, we collected scan frequency and stored tip counts per participant. Each week, the respondents received an email notification that new tips have been uploaded to their stickers. A preview of the stickers is available on Supplementary Figure S6.

### Econometric analysis

Our empirical strategy aims to estimate the causal effects of the two behavioral interventions (Nudge and Boost) on the electricity consumption of the participants over time. We adopt an intent-to-treat (ITT) approach, analyzing participants according to their randomly assigned condition regardless of subsequent engagement intensity. The ITT corresponds to the policy-relevant effect of offering the intervention to a target population. We analyze a panel of 140 participants with weekly electricity consumption observations spanning June 2023 to May 2024 (6,177 participant-week observations). Treatment timing varied slightly across participants depending on their registration date, but 84% of participants were enrolled by the end of 2023, ensuring that treatment onset clustered between calendar weeks 48 2023 and 6 2024. Concerns about bias in staggered two-way fixed-effects estimators^22^ are mitigated in our setting by (i) the inclusion of a never-treated control group, (ii) the narrow ten-week window over which treatment onset is distributed for the bulk of participants, and (iii) the absence of strong dynamic treatment-effect heterogeneity (see Supplementary Fig. S2). A robustness check excluding participants with fewer than 15 pre-treatment weekly observations yields qualitatively identical results (see Supplementary Table S13).

Let ${y}_{it}$ denote weekly electricity consumption (in kWh) of dwelling *i* in week *t*. Electricity consumption was measured at the dwelling level, as determined by the housing providers, metering infrastructure. Dwellings in our analysis sample range from single-occupancy studios to small shared apartments: 52% housed a single resident and 44% housed two residents, with the remaining 4% housing three or four (mean household size 1.5; see Supplementary Table S12). The dwelling is therefore the natural unit of measurement, and per-capita disaggregation was not feasible since individual consumption within multi-person dwellings is not separately metered. Occupancy is plausibly stable over the trial period (students typically did not change housing during the seven-month observation window) and is therefore absorbed, together with all other time-invariant dwelling characteristics, by the individual fixed effects in our preferred specification. Random assignment further produced balanced household sizes across conditions (Supplementary Table S12).

Participants were randomly assigned to one of three conditions: Control, Nudge, or Boost. We define treatment indicators as ${Nudge}_{i}=1$ if participant *i* was assigned to the Nudge condition and ${Boost}_{i}=1$ if assigned to Boost. The Control group serves as the base. The dummy ${AfterTreatment}_{it}=1$ for weeks after the intervention started for participant *i*, and 0 otherwise. Randomization produced groups that were comparable on most observed baseline characteristics. Supplementary Table S12 reports group means for pre-treatment electricity consumption, demographic characteristics, and baseline psychological measures, together with one-way ANOVA F-tests of equality across the three conditions. None of the tested baseline differences reaches conventional significance (smallest p = 0.125 for household size; baseline consumption: p = 0.80). The Boost group exhibits a modestly lower mean baseline consumption (20.9 vs. 22.0 kWh/week in the control group), which while not statistically significant motivates our preferred individual-fixed-effects specification below. We first estimate pooled OLS difference-in-differences models comparing average electricity consumption before and after treatment across the three experimental arms. The estimation equation is

$$ \begin{gathered} y_{it} = Nudge_{i} + \beta_{2} Boost_{i} + \beta_{3} AfterTreatment_{it} + \beta_{4} AfterTreatment_{it} \times Nudge_{i} \hfill \\ \quad \; + \beta_{5} AfterTreatment_{it} \times Boost_{i} + \gamma_{t} + \smallint_{it} , \hfill \\ \end{gathered} $$

where ${\gamma}_{t}$ are weekly dummies to control for common time trends such as holidays and seasonality. Standard errors ${\epsilon}_{it}$ are robust to heteroskedasticity. Results are reported in Fig.2 (blue coefficients) in the main text and Supplementary Table S1.

To account for potential baseline imbalances, especially the pre-treatment differences in the boost group, and to allow for unobserved heterogeneity, we estimate individual-level fixed-effects models:

$$ \begin{gathered} y_{it} = \beta_{3} AfterTreatment_{it} + \beta_{4} AfterTreatment_{it} \times Nudge_{i} + \beta_{5} AfterTreatment_{it} \times Boost_{i} \hfill \\ \quad \; + \gamma_{t} + \mu_{i} + u_{it} , \hfill \\ \end{gathered} $$

${\mu}_{i}$ where is the individual fixed effect accounting for all participant-specific factors that are constant over time. We use Driscoll-Kraay standard errors ^23^, which provide a non-parametric correction for arbitrary cross-sectional dependence across participants in the same week, together with corrections for serial correlation and heteroskedasticity. This approach accommodates potential spillovers among participants who shared accommodation facilities without requiring explicit specification of the spillover structure. Results are reported in Fig.2 (red coefficients) in the main text and Supplementary Table S2.

The primary estimates of interest in both models are ${\beta}_{4}$ and ${\beta}_{5}$ for the interactions between treatment assignment and the post-treatment period, i.e. ${AfterTreatment}_{it}\times {Nudge}_{i}$ and ${AfterTreatment}_{it}\times {Boost}_{i}$. Fixed-effects estimations provide our preferred specification due to their robustness to individual-specific confounders and intra-group correlation and are used for the moderation analyses.

To assess mechanisms, we analyze competence scores (variable ${z}_{it}$) elicited by energy-saving knowledge quizzes as secondary outcomes measured before and after the intervention. As this outcome is only elicited twice, we estimate both pooled OLS and fixed-effects models using the total time that participants spent interacting with the dashboard measured in seconds (variable ${TimeSpentWithDashboard}_{i}$) as additional control.

For pooled OLS the estimation equation is

$$ \begin{aligned} z_{it} = & \beta_{0} + \beta_{1} Nudge_{i} + \beta_{2} Boost_{i} + \beta_{3} AfterTreatment_{it} \\ & + \beta_{4} AfterTreatment_{it} \times Nudge_{i} + \beta_{5} AfterTreatment_{it} \times Boost_{i} \\ & + \delta TimeSpentWithDashboard_{i} + \in_{it} \\ \end{aligned} $$

Standard errors $\in_{it}$ are robust to heteroscedasticity. Results are reported in Fig. 6 (blue coefficients) in the main text and supplementary Table S6

The before-after fixed effects model is:

$$ \begin{gathered} z_{it} = \beta_{3} AfterTreatment_{it} + \beta_{4} AfterTreatment_{it} \times Nudge_{i} \hfill \\ \quad \; + \beta_{5} AfterTreatment_{it} \times Boost_{i} + \mu_{i} + u_{it} , \hfill \\ \end{gathered} $$

where ${\mu}_{i}$ is the individual fixed effect and standard errors ${u}_{it}$ are clustered at the participant level. Results are reported in Fig.4 (red coefficients) in the main text and Supplementary Table S9.

All graphs and regressions were produced with Stata version 18. To account for multiple hypothesis testing, we applied the Benjamini-Hochberg procedure ^24^ to control the false discovery rate (FDR) at 5%. Out of the ten pre-specified treatment comparisons, four remain statistically significant at the FDR-adjusted 5% level (adjusted p-values ≤ 0.05), and a fifth comparison is marginally significant (adjusted p = 0.061). We report both unadjusted and FDR-corrected p-values in Supplementary Table S11.

## Results

### Treatment effects on cumulated electricity use

We based our analyses on observations from 140 participants and 6,177 weekly electricity consumption measures from June 2023 to May 2024. The three experimental groups were comparable on baseline characteristics, with no statistically significant differences in pre-treatment electricity consumption, demographic variables, or pre-treatment psychological measures.

**Fig. 1:**
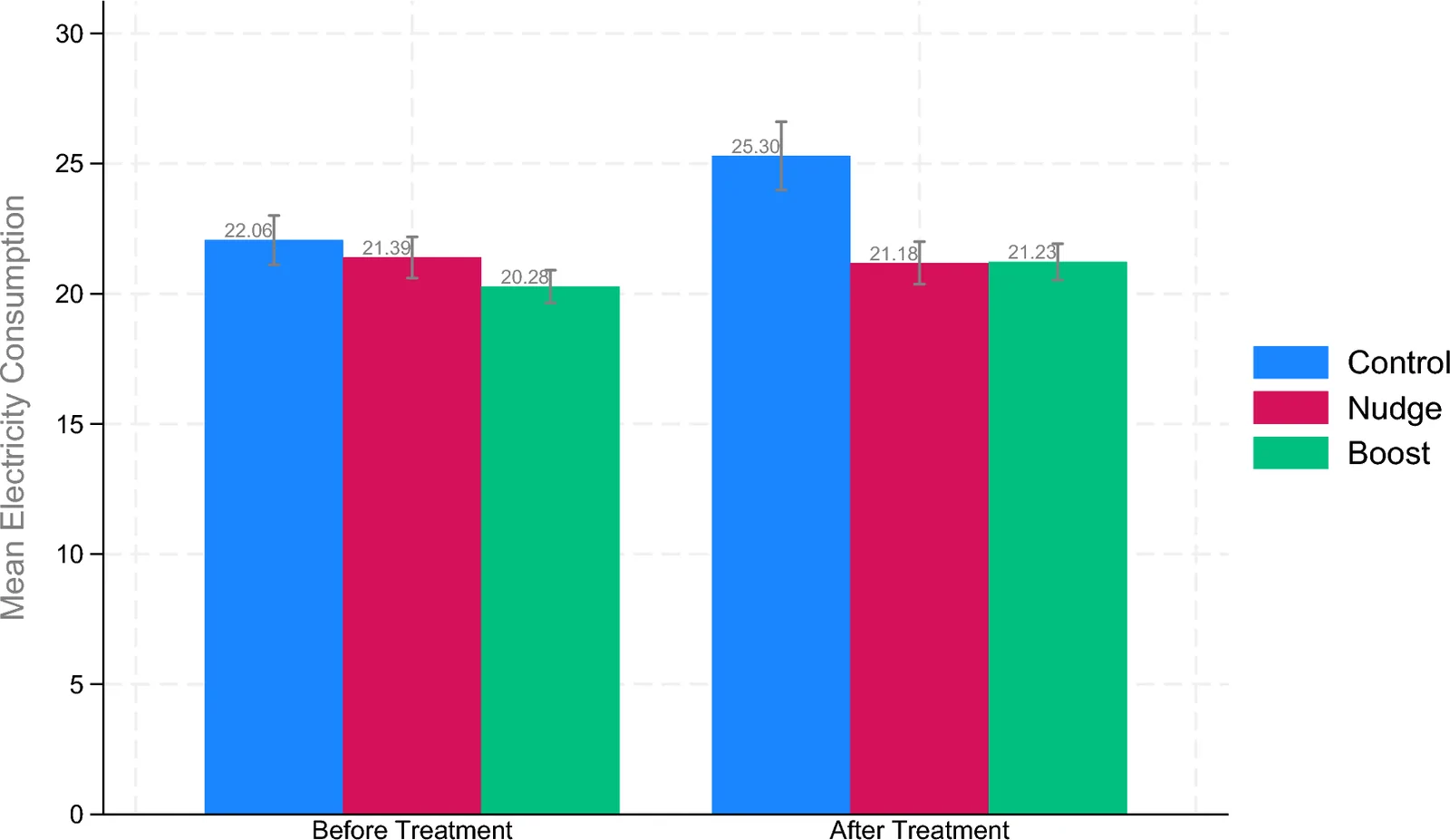
Mean cumulative weekly electricity consumption before and after treatment. Bar graphs show cumulative mean weekly electricity consumption in kWh and 95% confidence intervals in the treatment and control groups for the weeks before and the weeks after the treatment start.

As Fig. 1 demonstrates, participants in the control condition increased their electricity consumption after the treatment started. The pattern reflects a seasonal trend: Most participants started the treatment in December or early January, when it gets darker and colder than in the months preceding the treatment. As daylight time shrinks and temperatures plummet, the students tend to spend more time at home in the winter months and often use power-intensive space heaters to keep warm. Naturally, their electricity consumption goes up. The same seasonal pattern is visible in the available pre-experimental consumption data from the year preceding the trial (Supplementary Fig. S7). In contrast, in the same period the electricity consumption in the Nudge and Boost conditions remained without significant change.

As the treatment period coincided with winter, the estimated treatment effects should be interpreted as relative reductions compared with the control trend. As Fig. 1 shows, electricity consumption increased in the control condition after treatment onset, whereas consumption in the Nudge and Boost conditions remained comparatively stable. Thus, the interventions did not necessarily produce absolute within-group decreases from pre-treatment consumption levels. Rather, they attenuated the seasonal increase observed among untreated households. Statistically, this relative reduction is captured by the negative treatment-by-period interaction terms in the difference-in-differences models.

**Fig. 2:**
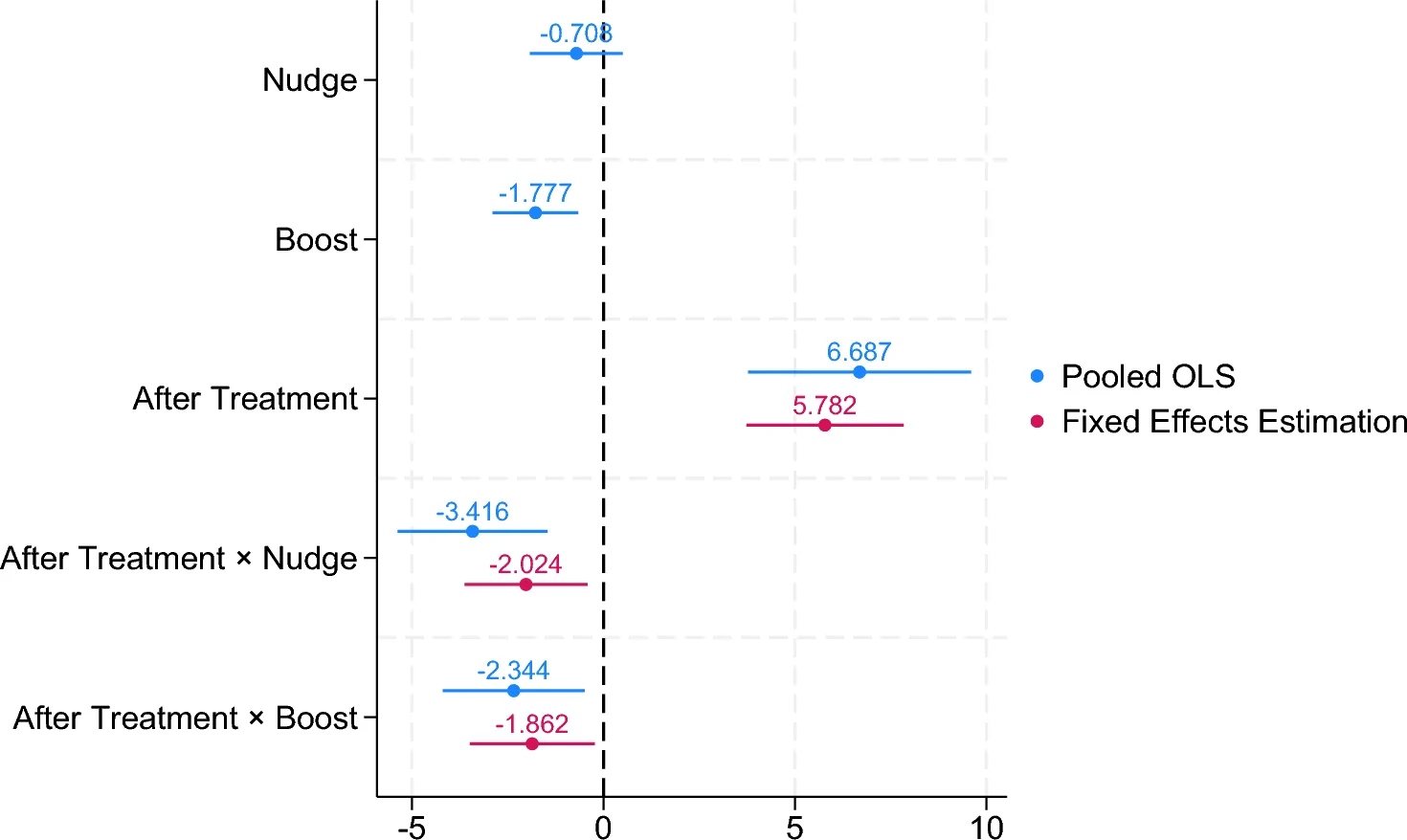
Estimation results for energy consumption. The blue graphs show coefficients and 95% confidence intervals from a pooled OLS estimation with robust standard errors. The red graphs show coefficients and 95% confidence intervals from fixed-effects estimation with Driscoll-Kraay standard errors. The dummies Nudge and Boost are dropped from the fixed effects estimation as they exhibit no time variation. Both estimations include weekly dummies to control for time trends that are not reported in the figure.

A pooled OLS difference-in-differences regression (Fig. 2, coefficients and robust standard errors in blue) shows that post-treatment, the Boost group used 2.34 kWh less per week (*p* = 0.013) on average in comparison to the control group, while the Nudge group reduced their electricity consumption by 3.42 kWh (*p* = 0.001). In contrast, participants in the control condition increased their electricity consumption by 6.687 kWh (p < 0.001) per week on average, compared to the pre-treatment period. However, the estimation also demonstrated that the Boost group exhibited pre-treatment consumption differences (Coef. = -1.777, p = 0.002), reflecting within-sample variation across participants that is not controlled for in this specification.

Therefore, we controlled for potential differences in group composition by involving individual fixed effects, where treatment effects are identified from within-participant variation. In addition, we allowed for correlations in the error term within and across participants. This is important in the context of our experiment: multiple participants lived in the same buildings, and it is possible that they communicated and influenced each other,s energy saving efforts. Individual-level fixed-effects regressions with robust Driscoll-Kraay standard errors (Fig. 2, in red) confirm that compared to the control group, the boosted participants significantly reduced their electricity consumption post-treatment (Boost × After: Coef. = -1.862, *p* = 0.026), as did the nudged (Nudge × After: Coef. = -2.024, *p* = 0.015). In the control group, the participants increased their energy use (After Treatment: Coef. = 5.782, p < 0.001) reflecting the seasonal trend. Complete regression results are reported in Supplementary Tables S1 and S2. A robustness check excluding the affected weeks 55–59 (data delivery problem and Christmas vacations, see also Fig. 4) yields quantitatively very similar estimates, showing that our findings are not driven by observations in the affected period (Supplementary Table S13).

To assess whether the relative impact of the interventions depends on baseline consumption levels, we additionally explored treatment effects separately for participants above and below the median of pre-treatment electricity use. Weeks 44 to 47 served as the pre-treatment period for this analysis, as no participants had been exposed to the intervention at that time. During these four weeks, the median electricity consumption was 20.6 kWh per week.

**Fig. 3:**
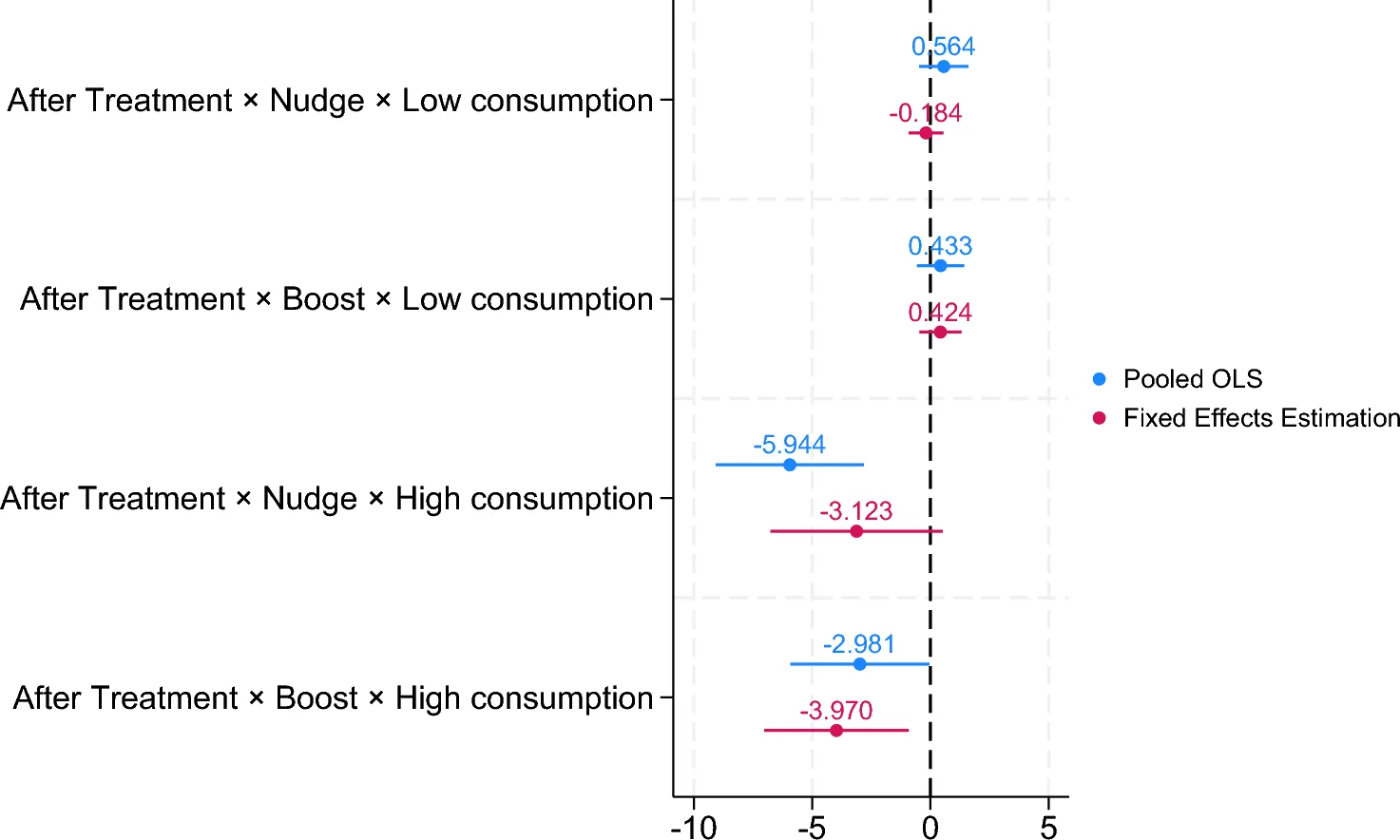
Estimation results for heterogeneity of treatment effects in prior consumption The blue graphs show coefficients and 95% confidence intervals from a pooled OLS estimation with robust standard errors. The red graphs show coefficients and 95% confidence intervals from fixed-effects estimation with Driscoll-Kraay standard errors. The graph only reports results for the relevant double and triple interactions. The dummies Nudge, Boost and Low consumption are dropped from the fixed effects estimation as they exhibit no time variation. Both estimations include weekly dummies to control for time trends that are not reported in the figure

Figure 3 presents the results of the heterogeneity analysis based on participants, pre-treatment consumption levels (complete regression results are reported in Supplementary Tables S3 and S4). The interaction terms *After Treatment × Nudge × Low Consumption* and *After Treatment × Boost × Low Consumption* indicate that participants with low baseline consumption did not respond to the interventions, likely because of limited potential for further reduction, or infrequent presence at home. The treatment effects appear to be driven by participants with high pre-treatment consumption, where the participants in the Boost treatment significantly reduced their electricity consumption. This is reflected in the significant triple interaction term for the Boost: *After Treatment × Boost × High Consumption* (β = –3.97, *p* = 0.01), and the marginally significant term for the Nudge: *After Treatment × Nudge × High Consumption* (β = –3.12, *p* = 0.09) in the fixed-effects estimation.

In sum, despite our expectations (H1), both the Boost and Nudge interventions were similarly effective in reducing electricity consumption during the study period. However, this effect was driven by participants with higher baseline usage. Within this group, only the Boost intervention yielded a statistically significant reduction, whereas the Nudge effect was inconclusive.

### Divergence between treatments over time

**Fig. 4:**
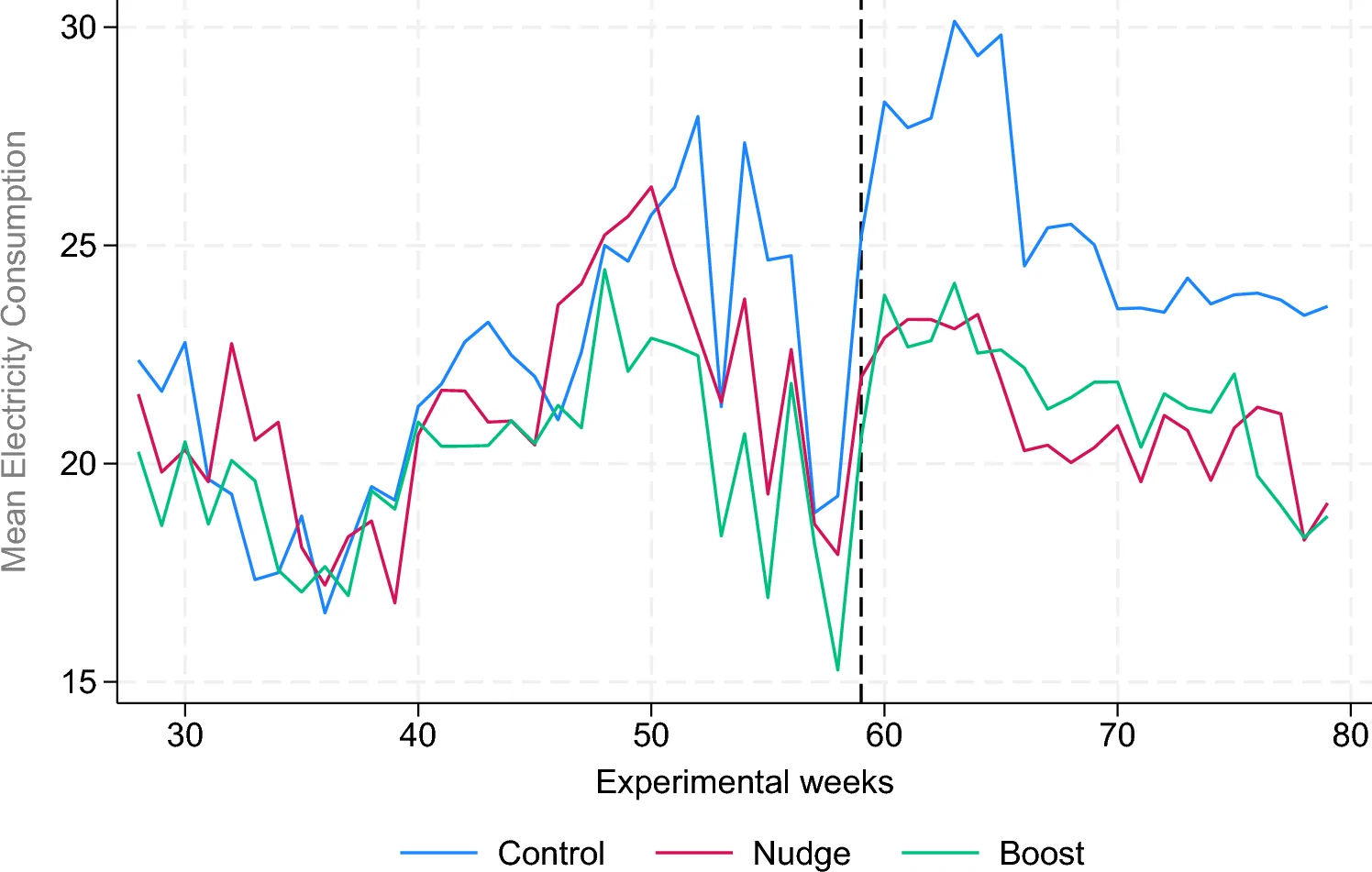
Energy consumption trajectories over experimental weeks. Line graphs show the course of mean electricity consumption per experimental week. The treatments started between weeks 55 and 65 corresponding to calendar weeks 48 in 2023 to 6 in 2024. 84% of participants registered until the end of 2023 (black dashed line). Between experimental weeks 55 and 59 there was a problem with the data delivery from the energy provider, and the Christmas vacations took place, when students typically visit their families. Both of these factors contributed to the drop in values during these weeks. A robustness check for our main regression excluding these weeks confirms that our main results are not affected (Supplementary Table S13).

Next, we examined whether the effects of the Boost and Nudge interventions diverged over the course of the experiment, as we would expect if boosting became increasingly effective due to progressing competence internalization (H3). A visual inspection of the weekly consumption trajectories (Fig. 4) reveals that this was not the case, since electricity use in both treatment groups followed highly parallel trends. The same holds true even if we split the groups into high and low pre-treatment consumers as in the previous section.

To quantify, we estimated treatment effects separately for three periods: weeks 0 to 5 after the treatment started, weeks 6 to 11 after the treatment started, and weeks 12 to 17 after the treatment started. The results are reported in Supplementary Figure S2 (see also Supplementary Tables S5-S7 there) and show that the treatment effects correspond to the average treatment effects in the first six weeks. After that, the effect of Nudge gets stronger in the middle six weeks, before the effects get statistically insignificant in the final six weeks. However, the differences between Nudge and Boost are statistically not significant with the lowest p-value of 0.22 for the middle six weeks. Thus, our segmented analysis suggests that there is no substantial divergence between the two treatments in any of these intervals.

## Progressive increase in Boost effectiveness

Next, we examined whether the Boost intervention led to progressively larger energy savings over time (H4), based on the assumption that competence-enhancing interventions generate cumulative behavioral effects. Our segmented analysis showed that the boost effect followed the predicted direction during the first 11 weeks but then weakened in the final period (as did the nudge effect). As such, the pattern is only partially consistent with a cumulative boost effect. The non-significant coefficient for weeks 11-17 points to a possible decay in engagement or treatment fatigue in the boost condition.

To further explore these patterns, we plotted the average weekly treatment engagement in both conditions. For the boosted participants, we calculated engagement as the mean weekly QR code scans (as scans were necessary to receive the new weekly tips). For the nudged participants, we took the mean weekly dashboard visits, where normative feedback was presented. In the boosted group, engagement with our energy saving tips peaked at about 2.5 months into the experiment. The peak was followed by a steady weekly decline until the end of May, when engagement dropped to less than one tip scan per week (see Supplementary Figure S3, panel A). For the nudged, engagement with the normative feedback declined initially until mid-February 2024 but remained relatively stable at around half a dashboard visit over time after that (Supplementary Figure S3, panel B).

## Competence enhancement and mechanisms

**Fig. 5:**
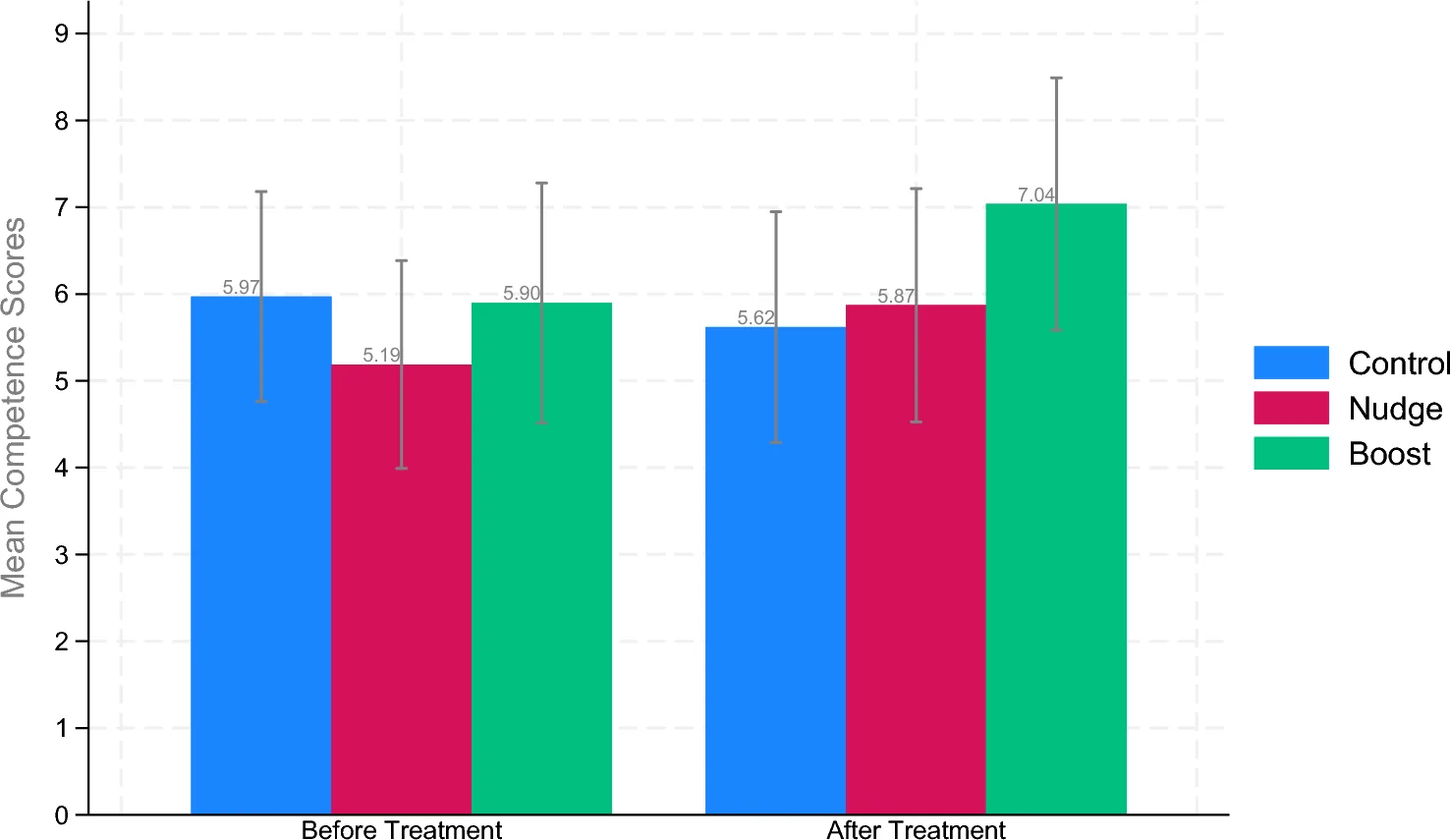
Mean competence scores before and after treatment. Bar graphs show mean competence scores and 95% confidence intervals in the treatment and control groups from the energy competence quizzes conducted before the treatment started and after the experiment concluded.

**Fig. 6:**
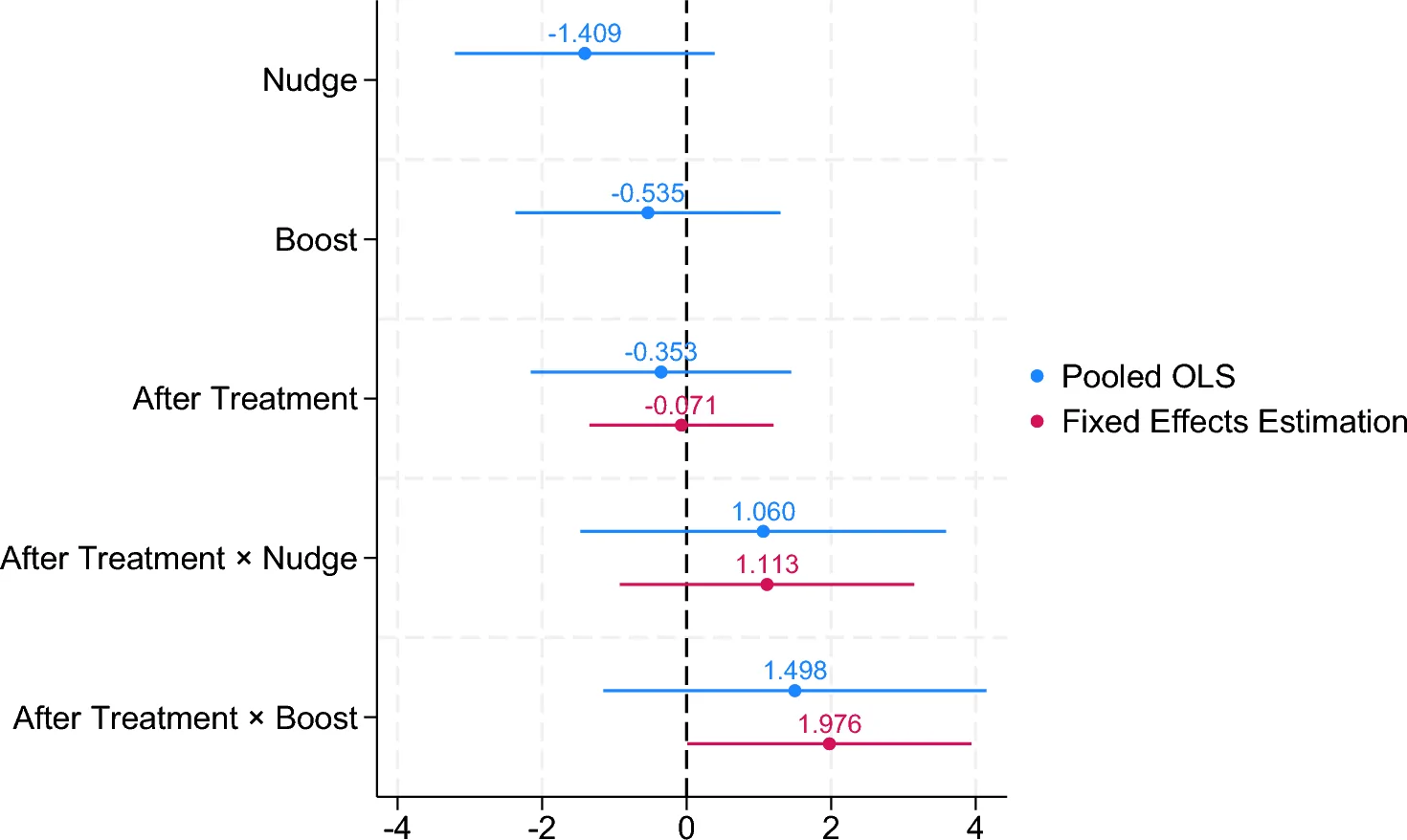
Estimation results for competencies. The blue graphs show coefficients and 95% confidence intervals from a pooled OLS estimation with robust standard errors. The red graphs show coefficients and 95% confidence intervals from fixed-effects estimation with standard errors clustered at participant levels. The dummies Nudge and Boost are dropped from the fixed effects estimation as they exhibit no time variation. Both estimations include a control for the total time that participants spent with their dashboards (personal home pages, please see Methods for more information), which are not reported in the graph.

To test whether the boost intervention improved energy saving competences (H2), we analyzed the scores from energy competence quizzes conducted before and after the experimental period. The mean scores shown in Fig. 5 indicate no change in the control group and a modest increase in competence scores for the Nudge and Boost treatments. While the difference between the nudged and the boosted groups did not reach statistical significance, only the participants in the Boost treatment marginally increased their competence scores by about 2 points (*p* = 0.05) relative to the control group (see fixed effects estimation in Fig. 6 and Supplementary Table S9). When corrected for multiple hypotheses testing, the significance becomes marginal (*p* = 0.06, see Supplementary Table S11 for the adjusted values per hypothesis). Thus, H2 received only partial support: the boost showed marginal evidence of competence improvement relative to control, but competence gains were not clearly unique to the boost condition.

Although we pre-registered a mediation analysis to test whether improvements in energy-saving competences mediated the effect of our interventions on electricity consumption, only the Boost intervention led to a modest increase in competence scores. Given such limited first-stage effect, the assumptions required for mediation analysis were not met. Therefore, as indicated in the pre-registration, we redirected our focus to potential moderation effects, starting with baseline competences and pro-environmental motivation.

Previous literature suggests that competence alone may not be sufficient to trigger sustained behavioral change; rather, its impact may depend on motivational factors such as environmental motivation^4^. Based on this rationale, we estimated treatment effects for four subgroups defined by combinations of high versus low pre-treatment competence and high versus low environmental motivation. Both variables were dichotomized at the sample median to create binary indicators for high and low levels, respectively.

Supplementary Figure S4 reports the results of this double-moderator analysis using individual-level fixed-effects regressions (see also Supplementary Table S10). They indicate that energy savings occurred only among participants who scored above the median on both competence and environmental motivation. In contrast, neither high competence or high motivation alone was associated with statistically significant reductions in electricity consumption. The pattern suggests that our behavioral interventions were effective for participants who were both capable and motivated to act on the provided information.

## Discussion

In a randomized field experiment, we demonstrated that both nudges (normative feedback) and boosts (competence-enhancing tips) significantly reduced household electricity consumption relative to the control trend. Substantively, both interventions prevented the seasonal increase in electricity use observed among untreated households. Collectively, the interventions corresponded to meaningful avoided consumption (~7 MWh) over the 24-week trial, demonstrating the practical potential of both behavioral approaches for sustainable energy policy. Achieved without hardware changes or financial incentives, these reductions show that simple, scalable communications can meaningfully trim residential demand.

We had hypothesized that participants in the boost treatment would consume less electricity than participants in the nudge condition over the intervention period (H1). Instead, both treatments significantly reduced electricity consumption relative to the control group, but their effects were statistically indistinguishable from each other. Hence, we demonstrated that within a student residential context, a competence-enhancing boost can deliver energy savings comparable to those of already well-established intervention strategies^15,20^.

We also anticipated that the difference in energy consumption between the boost and nudge conditions would become greater toward the end of the treatment period (H3), and that the boosted participants will consume less electricity over time (H4). These hypotheses were not supported, as the consumption trajectories in the two treatments remained largely parallel. Our expectations were based on the assumption that competence acquisition would accumulate gradually in the boost treatment and will translate into increasingly larger behavioral effects. Instead, the data showed only marginally higher pre-post competence scores for the boosted group relative to control (in partial support to H2), while the nudge group also exhibited a smaller descriptive increase. Two possible explanations emerge. First, the competence acquisition in the boost treatment might not have been as gradual as predicted, as hinted by the gradual drop in treatment engagement in the boosted group. As the boosted participants accessed fewer tips over time, competence buildup might not have been consistent enough to result in increased savings. Second, competence acquisition might not have directly translated into energy saving behavior. This is consistent with a broader literature showing that knowledge, competence, and awareness do not automatically produce pro-environmental behavior. The relationship between what people know and what they do is often indirect, moderated by motivation, opportunity, habits, and contextual constraints^5,25,26^. Even action-related knowledge must still be activated in concrete situations^27^. Hence, in our study, the QR-code tips may have increased residents, energy-saving competence, but this competence likely required sustained engagement and motivation before generating progressive energy savings.

Our heterogeneity analyses further qualified the treatment effects. High baseline consumers were responsible for most of the observed reductions, and within this group the boost effect was statistically significant whereas the nudge effect was inconclusive. Hence, the boost helped where help was needed the most: high-consumption households plausibly need more actionable saving opportunities. At the same time, our moderator analysis suggested that baseline consumption was not the only relevant source of heterogeneity. Energy savings occurred primarily among participants who were both relatively competent and motivated. The finding is consistent with previous evidence that energy-saving boosts are most effective among highly motivated participants^4^.

Several contributions set our study apart from previous work. First, we provide rare comparative field evidence on two theoretically distinct behavioral intervention strategies for sustainable consumption: a normative-feedback nudge and a competence-enhancing boost. Only a small number of existing studies directly contrast energy-saving strategies with each other. Within this literature, some studies rely on online simulated tasks^28^, others examine groups who self-selected into program participation^29^, and others focus on shorter field intervention periods^30^. Against this background, our study adds a 24-week randomized field experiment with metered electricity consumption as the behavioral outcome. The two studies most similar in design are Lazaric and Toumi^4^ and Paunov and Grüne-Yanoff^6^, both of which provide important field evidence, but are based on smaller samples. Our study therefore adds a comparatively larger randomized field test of nudging and boosting in a real residential setting. Furthermore, we contribute mechanism-relevant evidence by measuring not only energy use, but also competence and engagement, thereby assessing whether the interventions reflected their theoretical accounts.

Several limitations can be considered when interpreting our results. First, the student housing sample we used may limit the generalizability of the findings. Second, the proximity of the housing complexes might have led to information sharing among participants, possibly undermining the treatment differences. This is a speculative claim, but is particularly relevant for our competence measure, where both treatment groups showed some improvement. Third, although the 24-week duration is relatively long compared with other intervention studies, it may still have been insufficient for robust competence development and routinization. As engagement with the energy saving tips declined over time, the boost may not have provided enough sustained exposure for competence gains to accumulate into stronger behavioral effects. Fourth, while treatment assignment was randomized immediately upon enrolment, participation in the study itself was voluntary. Hence, our sample may overrepresent students who were more interested in sustainability or research participation. Such potential self-selection does not undermine the internal comparison between randomized treatment groups, but it may limit the extent to which the effects generalize to less motivated residents.

Finally, some design and interpretation issues should be kept in mind. Treatment start dates were slightly staggered as participants enrolled dynamically over time. Our models account for this by defining the post-treatment period at the participant level and by including weekly fixed effects, but the staggered implementation nevertheless means that treatment exposure did not begin on the same calendar week for all participants. In addition, our competence findings should be interpreted cautiously: the boost-related competence increase was marginal after correction for multiple testing, and the nudge group also showed a smaller descriptive increase. Finally, the treatment effects were not equally stable across the whole intervention period, with stronger effects in the early and middle phases and weaker evidence in the final weeks.

## Conclusion

Despite limitations, our findings have clear practical implications for the use of boosts and nudges for policymakers. First, they demonstrate that competence-enhancing interventions can be at least as promising as their nudge-based counterparts for attenuating seasonal increases in electricity use. Second, boosts can be especially useful for targeting high-consumption households, where efficiency gains are needed most. Third, the choice of intervention should depend on the intended mechanism. Normative feedback appears useful when the aim is to sustain attention to consumption through repeated social comparison, whereas boosts may be especially appropriate when residents lack practical knowledge about how to save energy. However, our results also suggest that persistent effects from boosts may be improved by using delivery systems that sustain participants, engagement, such as repeated prompts and visually salient delivery. Such designs could simultaneously keep energy use salient and provide residents with the practical skills needed to act on that motivation.

## Supplementary Information

Supplementary Information 1.

Supplementary Information 2.

Supplementary Information 3.

Supplementary Information 4.

Supplementary Information 5.

Supplementary Information 6.

Supplementary Information 7.

Supplementary Information 8.

## Data Availability

The raw consumption and survey data is available in the OSF repository here: https://osf.io/fwesh/files

## Code availability

The STATA script required for all analyses is available here: https://osf.io/fwesh/files

## Declarations

### Pre-registration

This experiment was pre-registered on OSF Registries on February 13, 2024. The pre-registration is available at: https://doi.org/10.17605/OSF.IO/7FW46
