# Supplementary material for: A randomized field experiment comparing nudges and boosts for household electricity conservation in student housing
Source: Sci Rep. 2026 Sep 3;16:27689. doi: 10.1038/s41598-026-69386-6 (PMC13542069; doi:10.1038/s41598-026-69386-6)
Supplement: Supplementary file 1 [file 41598_2026_69386_MOESM2_ESM.pdf]

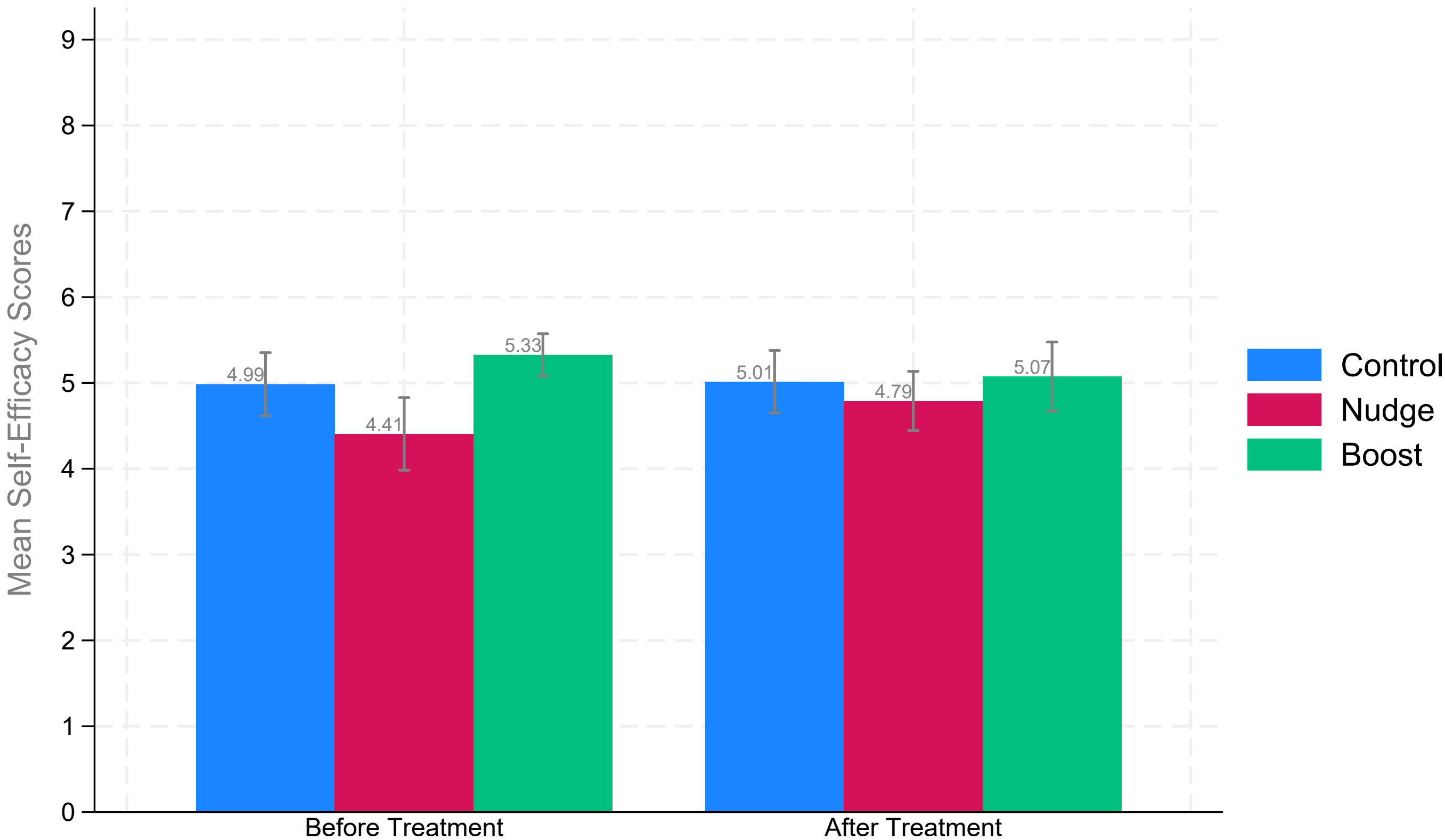

Mean Self-Efficacy Scores
0
1
2
3
4
5
6
7
8
9
4.99
4.41
5.33
5.01
4.79
5.07
Before Treatment
After Treatment
Control
Nudge
Boost
