# Supplementary material for: A randomized field experiment comparing nudges and boosts for household electricity conservation in student housing
Source: Sci Rep. 2026 Sep 3;16:27689. doi: 10.1038/s41598-026-69386-6 (PMC13542069; doi:10.1038/s41598-026-69386-6)
Supplement: Supplementary file 2 [file 41598_2026_69386_MOESM3_ESM.pdf]

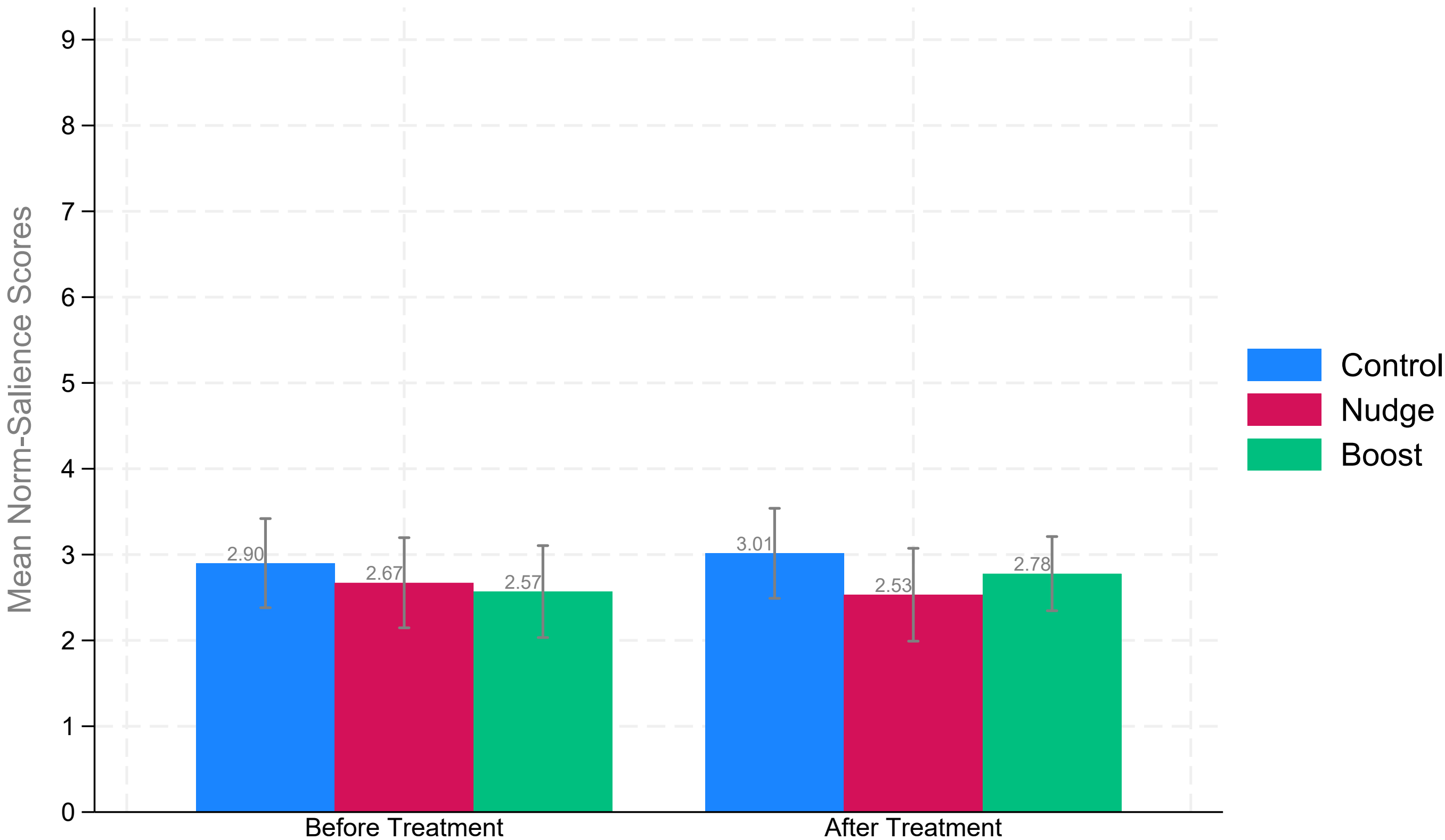

Mean Norm-Salience Scores
9
8
7
6
5
4
3
2
1
0
2.90
2.67
2.57
3.01
2.53
2.78
Before Treatment
After Treatment
Control
Nudge
Boost
