# Supplementary material for: A randomized field experiment comparing nudges and boosts for household electricity conservation in student housing
Source: Sci Rep. 2026 Sep 3;16:27689. doi: 10.1038/s41598-026-69386-6 (PMC13542069; doi:10.1038/s41598-026-69386-6)
Supplement: Supplementary file 3 [file 41598_2026_69386_MOESM4_ESM.pdf]

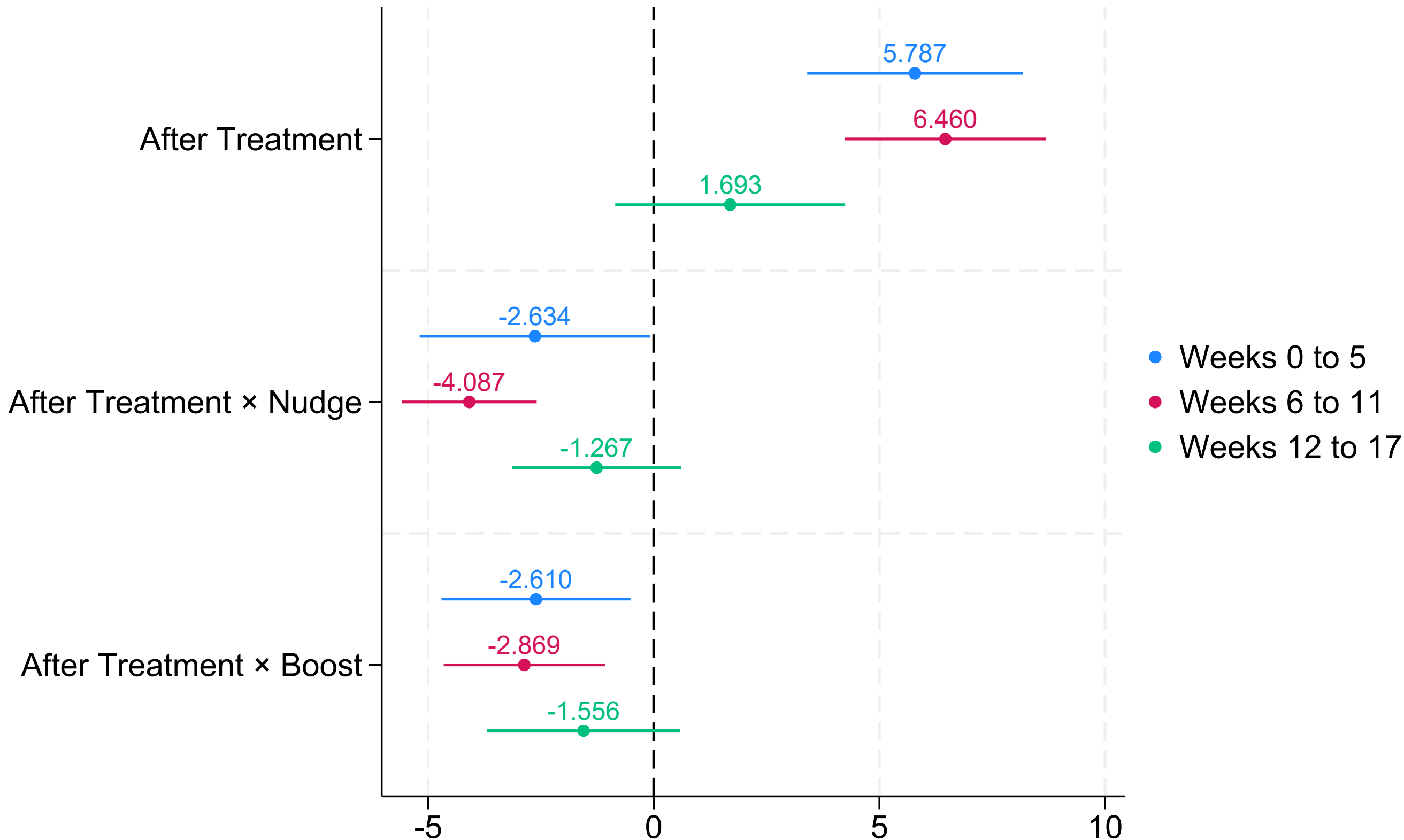
After Treatment
After Treatment × Nudge
After Treatment × Boost
5.787
6.460
1.693
-2.634
-4.087
-1.267
-2.610
-2.869
-1.556
-5
0
5
10
Weeks 0 to 5
Weeks 6 to 11
Weeks 12 to 17
