# Supplementary material for: A randomized field experiment comparing nudges and boosts for household electricity conservation in student housing
Source: Sci Rep. 2026 Sep 3;16:27689. doi: 10.1038/s41598-026-69386-6 (PMC13542069; doi:10.1038/s41598-026-69386-6)
Supplement: Supplementary file 4 [file 41598_2026_69386_MOESM5_ESM.pdf]

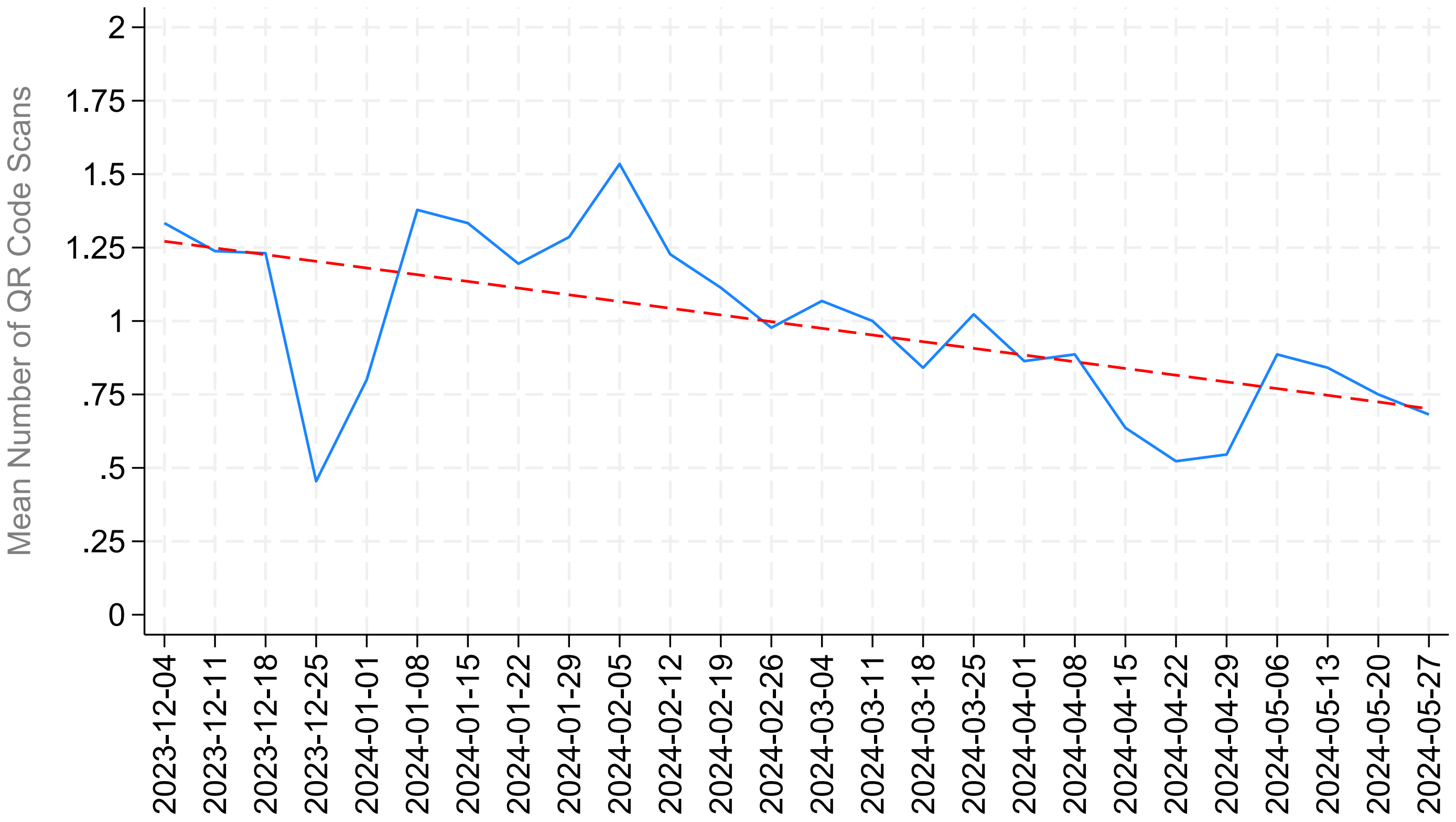

Mean Number of QR Code Scans
0
.25
.5
.75
1
1.25
1.5
1.75
2
2023-12-04
2023-12-11
2023-12-18
2023-12-25
2024-01-01
2024-01-08
2024-01-15
2024-01-22
2024-01-29
2024-02-05
2024-02-12
2024-02-19
2024-02-26
2024-03-04
2024-03-11
2024-03-18
2024-03-25
2024-04-01
2024-04-08
2024-04-15
2024-04-22
2024-04-29
2024-05-06
2024-05-13
2024-05-20
2024-05-27
