# Supplementary material for: A randomized field experiment comparing nudges and boosts for household electricity conservation in student housing
Source: Sci Rep. 2026 Sep 3;16:27689. doi: 10.1038/s41598-026-69386-6 (PMC13542069; doi:10.1038/s41598-026-69386-6)
Supplement: Supplementary file 6 [file 41598_2026_69386_MOESM7_ESM.pdf]

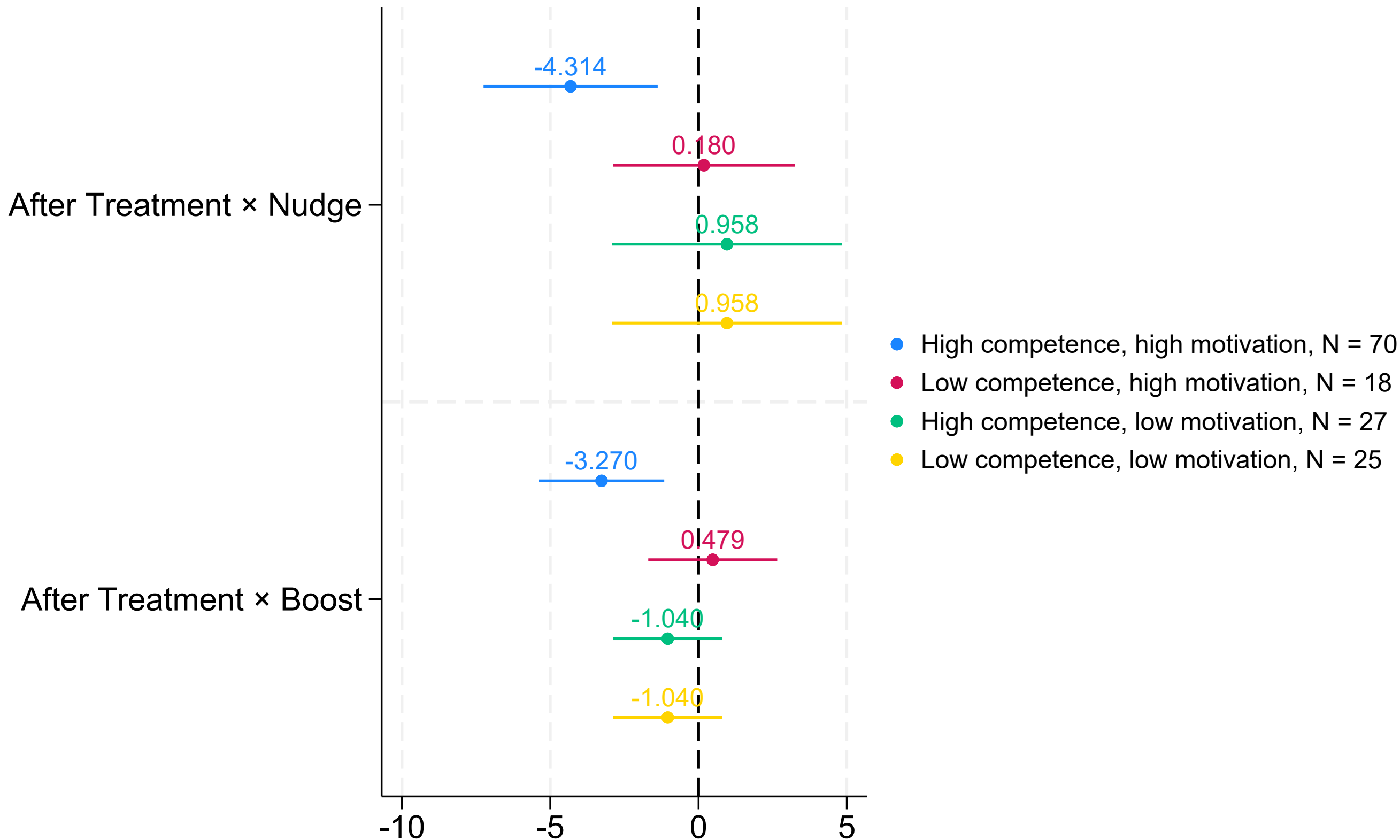
After Treatment × Nudge
After Treatment × Boost
-4.314
0.180
0.958
0.958
-3.270
0.479
-1.040
-1.040
-10
-5
0
5
High competence, high motivation, N = 70
Low competence, high motivation, N = 18
High competence, low motivation, N = 27
Low competence, low motivation, N = 25
