# Supplementary material for: A randomized field experiment comparing nudges and boosts for household electricity conservation in student housing
Source: Sci Rep. 2026 Sep 3;16:27689. doi: 10.1038/s41598-026-69386-6 (PMC13542069; doi:10.1038/s41598-026-69386-6)
Supplement: Supplementary file 7 [file 41598_2026_69386_MOESM8_ESM.pdf]

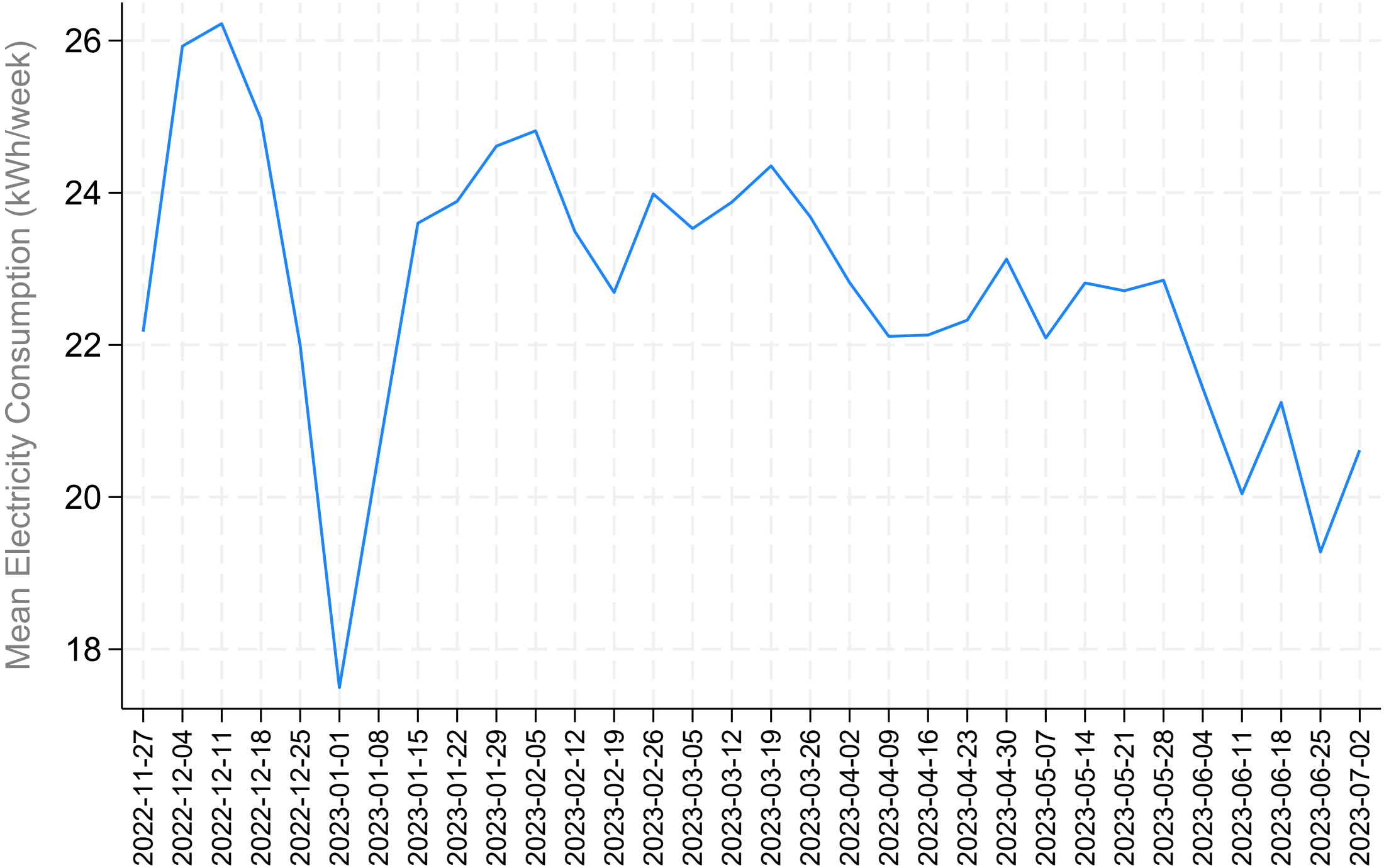

Mean Electricity Consumption (kWh/week)
26
24
22
20
18
2022-11-27
2022-12-04
2022-12-11
2022-12-18
2022-12-25
2023-01-01
2023-01-08
2023-01-15
2023-01-22
2023-01-29
2023-02-05
2023-02-12
2023-02-19
2023-02-26
2023-03-05
2023-03-12
2023-03-19
2023-03-26
2023-04-02
2023-04-09
2023-04-16
2023-04-23
2023-04-30
2023-05-07
2023-05-14
2023-05-21
2023-05-28
2023-06-04
2023-06-11
2023-06-18
2023-06-25
2023-07-02
