# Supplementary material for: A randomized field experiment comparing nudges and boosts for household electricity conservation in student housing
Source: Sci Rep. 2026 Sep 3;16:27689. doi: 10.1038/s41598-026-69386-6 (PMC13542069; doi:10.1038/s41598-026-69386-6)
Supplement: Supplementary file 8 [file 41598_2026_69386_MOESM1_ESM.docx]

**Supplementary Information**

**Behavioral interventions for sustainable consumption: comparing nudges and boosts in a student residential context**

Yavor Paunov, Till Grüne-Yanoff, Caterina Marchionni and Claudia Schwirplies

**Regression Tables**

Table S1: Pooled OLS regression results reported in Fig.2

| Consumption | Coef. | | St.Err. | t-value | | p-value | [95% Conf | | | Interval] | | | Sig |  |  |
| --- | --- | --- | --- | --- | --- | --- | --- | --- | --- | --- | --- | --- | --- | --- | --- |
| After Treatment | 6.687 | | 1.484 | 4.51 | | .000 | 3.778 | | | 9.596 | | | *** |  |  |
| Nudge | -.708 | | .618 | -1.14 | | .253 | -1.920 | | | .505 | | |  |  |  |
| Boost | -1.777 | | .570 | -3.12 | | .002 | -2.893 | | | -.660 | | | *** |  |  |
| After Treatment × Nudge | -3.416 | | 1.000 | -3.42 | | .001 | -5.376 | | | -1.456 | | | *** |  |  |
| After Treatment × Boost | -2.344 | | .946 | -2.48 | | .013 | -4.198 | | | -.489 | | | ** |  |  |
| Constant | 22.240 | | 1.089 | 20.42 | | .000 | 20.105 | | | 24.375 | | | *** |  |  |
| Weekly fixed effects not reported | | | | | | | | | | | | | |  |  |
| R-squared | | 0.033 | | Number of obs . | | | | 6177 | | |  | | | |  |
| F-test | | 3.369 | | Prob > F | | | | 0.000 | | |  | | | |  |
| Akaike crit. (AIC) | | 50977.617 | | | Bayesian crit. (BIC) | | | | 51361.146 | | |  | | | |
| Notes: Coefficients, robust standard errors and 95% confidence intervals from a pooled OLS estimation. The dependent variable is electricity consumption in kWh. Nudge and Boost are dummy variables equal to 1 if the user was in the respective treatment group. After Treatment is a dummy equal to 1 after the treatment started for the respective participant. Estimation includes weekly dummies to control for time trends that are not reported in the Table. *** p<.01, ** p<.05, * p<.1 | | | | | | | | | | | | | |  |  |

Table S2: Fixed effects regression results with Driscoll-Kraay standard errors reported in Fig.2

| Consumption | Coef. | | Drisc/Kraay std. err. | | t-value | p-value | | | [95% Conf. | | | Interval] | | | Sig. |  |  |
| --- | --- | --- | --- | --- | --- | --- | --- | --- | --- | --- | --- | --- | --- | --- | --- | --- | --- |
| After Treatment | 5.782 | | 1.022 | | 5.660 | 0.000 | | | 3.730 | | | 7.834 | | | *** |  |  |
| After Treatment × Nudge | -2.024 | | 0.800 | | -2.530 | 0.015 | | | -3.630 | | | -0.417 | | | ** |  |  |
| After Treatment × Boost | -1.862 | | 0.812 | | -2.290 | 0.026 | | | -3.493 | | | -0.232 | | | ** |  |  |
| Constant | 20.606 | | 0.254 | | 81.030 | 0.000 | | | 20.095 | | | 21.117 | | | *** |  |  |
| Within R-squared | | 0.0551 | | Number of obs. (part.) | | | 6177 (140) | | |  | | |  | | | |  |
| F-test | | 406279.36 | | Prob > F | | | 0.000 | | |  | | |  | | | |  |
| Week fixed effects | | YES | | User fixed effects | | | | Yes | | |  | | |  | | | |

Notes: Coefficients, standard errors and 95% confidence intervals from fixed-effects estimation with Driscoll-Kraay standard errors. The dependent variable is electricity consumption in kWh. Nudge and Boost are dummy variables which are 1 if the user was in the respective treatment group. After Treatment is a dummy equal to 1 after the treatment started for the respective participant. The dummies Nudge and Boost are dropped from the fixed effects estimation as they exhibit no time variation. Estimation includes weekly dummies to control for time trends that are not reported in the Table. *** p<.01, ** p<.05, * p<.1

Table S3: Pooled OLS regression results reported in Fig.3

| Consumption | Coef. | | St.Err. | t-value | | p-value | [95% Conf | | Interval] | Sig | |  |
| --- | --- | --- | --- | --- | --- | --- | --- | --- | --- | --- | --- | --- |
| After Treatment | 3.504 | | 1.164 | 3.01 | | .003 | 1.223 | | 5.786 | *** | |  |
| Nudge | 1.204 | | .371 | 3.25 | | .001 | .477 | | 1.93 | *** | |  |
| Boost | 1.218 | | .368 | 3.32 | | .001 | .498 | | 1.939 | *** | |  |
| After Treatment × Nudge | .564 | | .531 | 1.06 | | .288 | -.477 | | 1.604 |  | |  |
| After Treatment × Boost | .433 | | .509 | 0.85 | | .395 | -.565 | | 1.431 |  | |  |
| High | 15.371 | | .76 | 20.23 | | 0 | 13.881 | | 16.861 | *** | |  |
| After Treatment × High | 4.47 | | 1.272 | 3.51 | | 0 | 1.975 | | 6.964 | *** | |  |
| Nudge × High | -2.419 | | 1 | -2.42 | | .016 | -4.38 | | -.458 | ** | |  |
| Boost × High | -3.89 | | .925 | -4.21 | | 0 | -5.704 | | -2.077 | *** | |  |
| After Treatment × Nudge × High | -5.944 | | 1.599 | -3.72 | | 0 | -9.078 | | -2.811 | *** | |  |
| After Treatment × Boost × High | -2.981 | | 1.5 | -1.99 | | .047 | -5.922 | | -.04 | ** | |  |
| Constant | 12.744 | | .745 | 17.11 | | 0 | 11.284 | | 14.204 | *** | |  |
| Mean dependent var | | 21.979 | | | SD dependent var | | | 15.103 | | |  |  |
| R-squared | | 0.257 | | | Number of obs | | | 6177 | | |  |  |
| F-test | | 46.078 | | | Prob > F | | | 0.000 | | |  |  |
| Akaike crit. (AIC) | | 49356.543 | | | Bayesian crit. (BIC) | | | 49780.444 | | |  |  |
|  | | | | | | | | | | | | |

Notes: Coefficients, robust standard errors and 95% confidence intervals from a pooled OLS estimation. The dependent variable is electricity consumption in kWh. Nudge and Boost are dummy variables equal to 1 if the user was in the respective treatment group. After Treatment is a dummy equal to 1 after the treatment started for the respective participant. High is a dummy variable equal to 1 if the participant consumed above median in weeks 44 to 47 prior treatment. Estimation includes weekly dummies to control for time trends that are not reported in the Table. *** p<.01, ** p<.05, * p<.1

Table S4: Fixed effects regression results with Driscoll-Kraay standard errors reported in Fig.3

| Consumption | Coef. | St. Err. | | t-value | p-value | | | [95% Conf | | | Interval] | | | Sig. | | |
| --- | --- | --- | --- | --- | --- | --- | --- | --- | --- | --- | --- | --- | --- | --- | --- | --- |
| After Treatment | 3.181 | 0.967 | | 3.290 | 0.002 | | | 1.240 | | | 5.122 | | | *** | | |
| After Treatment × Nudge | -0.184 | 0.365 | | -0.500 | 0.617 | | | -0.917 | | | 0.550 | | |  | | |
| After Treatment × Boost | 0.424 | 0.443 | | 0.960 | 0.343 | | | -0.465 | | | 1.313 | | |  | | |
| After Treatment × High | 4.369 | 1.638 | | 2.670 | 0.010 | | | 1.080 | | | 7.658 | | | *** | | |
| After Treatment × Nudge × High | -3.123 | 1.814 | | -1.720 | 0.091 | | | -6.765 | | | 0.519 | | | * | | |
| After Treatment × Boost × High | -3.970 | 1.520 | | -2.610 | 0.012 | | | -7.022 | | | -0.919 | | | ** | | |
| Constant | 20.635 | 0.279 | | 73.910 | 0.000 | | | 20.075 | | | 21.195 | | | *** | | |
| Within R-squared | 0.059 | | Number of obs. (users) | | | 6177 (140) | | |  | | |  | | |  |  |
| F-test | 668147.09 | | Prob > F | | | 0.000 | | |  | | |  | | |  |  |
| Week fixed effects | Yes | | User fixed effects | | | | Yes | | |  | | |  | | |  |

Notes: Coefficients, standard errors and 95% confidence intervals from fixed-effects estimation with Driscoll-Kraay standard errors. The dependent variable is electricity consumption in kWh. Nudge and Boost are dummy variables which are 1 if the user was in the respective treatment group. After Treatment is a dummy equal to 1 after the treatment started for the respective participant. High is a dummy variable equal to 1 if the participant consumed above median in weeks 44 to 47 prior treatment. The dummies Nudge, Boost and High are dropped from the fixed effects estimation as they exhibit no time variation. Estimation includes weekly dummies to control for time trends that are not reported in the Table. *** p<.01, ** p<.05, * p<.1

Table S5: Fixed effects regression results for weeks 0 to 5 reported in Fig.S2 (see Figures below)

| Consumption | Coef. | St. Err. | | t-value | p-value | | | [95% Conf | | | Interval] | | | Sig. | | |
| --- | --- | --- | --- | --- | --- | --- | --- | --- | --- | --- | --- | --- | --- | --- | --- | --- |
| After Treatment | 5.787 | 1.183 | | 4.890 | 0.000 | | | 3.400 | | | 8.175 | | | *** | | |
| After Treatment × Nudge | -2.634 | 1.266 | | -2.080 | 0.044 | | | -5.188 | | | -0.079 | | | ** | | |
| After Treatment × Boost | -2.610 | 1.037 | | -2.520 | 0.016 | | | -4.704 | | | -0.516 | | | ** | | |
| Constant | 20.304 | 0.301 | | 67.460 | 0.000 | | | 19.696 | | | 20.911 | | | *** | | |
| Within R-squared | 0.076 | | Number of obs. (users) | | | 3900 (140) | | |  | | |  | | |  |  |
| F-test | 36646018.60 | | Prob > F | | | 0.000 | | |  | | |  | | |  |  |
| Week fixed effects | Yes | | User fixed effects | | | | Yes | | |  | | |  | | |  |

Notes: Coefficients, standard errors and 95% confidence intervals from fixed-effects estimation with Driscoll-Kraay standard errors. The dependent variable is electricity consumption in kWh. Nudge and Boost are dummy variables which are 1 if the user was in the respective treatment group. After Treatment is a dummy equal to 1 after the treatment started for the respective participant. The dummies Nudge and Boost are dropped from the fixed effects estimation as they exhibit no time variation. Estimation includes weekly dummies to control for time trends that are not reported in the Table. *** p<.01, ** p<.05, * p<.1

Table S6: Fixed effects regression results for weeks 6 to 11 reported in Fig.S2

| Consumption | Coef. | St. Err. | | t-value | p-value | | | [95% Conf | | | Interval] | | | Sig. | | |
| --- | --- | --- | --- | --- | --- | --- | --- | --- | --- | --- | --- | --- | --- | --- | --- | --- |
| After Treatment | 6.460 | 1.110 | | 5.820 | 0.000 | | | 4.228 | | | 8.692 | | | *** | | |
| After Treatment × Nudge | -4.087 | 0.742 | | -5.510 | 0.000 | | | -5.578 | | | -2.595 | | | *** | | |
| After Treatment × Boost | -2.869 | 0.888 | | -3.230 | 0.002 | | | -4.655 | | | -1.084 | | | *** | | |
| Constant | 20.335 | 0.334 | | 60.910 | 0.000 | | | 19.663 | | | 21.006 | | | *** | | |
| Within R-squared | 0.072 | | Number of obs. (users) | | | 3903 (140) | | |  | | |  | | |  |  |
| F-test | 5485533.27 | | Prob > F | | | 0.000 | | |  | | |  | | |  |  |
| Week fixed effects | Yes | | User fixed effects | | | | Yes | | |  | | |  | | |  |

Notes: Coefficients, standard errors and 95% confidence intervals from fixed-effects estimation with Driscoll-Kraay standard errors. The dependent variable is electricity consumption in kWh. Nudge and Boost are dummy variables which are 1 if the user was in the respective treatment group. After Treatment is a dummy equal to 1 after the treatment started for the respective participant. The dummies Nudge and Boost are dropped from the fixed effects estimation as they exhibit no time variation. Estimation includes weekly dummies to control for time trends that are not reported in the Table. *** p<.01, ** p<.05, * p<.1

Table S7: Fixed effects regression results for weeks 12 to 17 reported in Fig.S2

| Consumption | Coef. | St. Err. | | t-value | p-value | | | [95% Conf | | | Interval] | | | Sig. | | |
| --- | --- | --- | --- | --- | --- | --- | --- | --- | --- | --- | --- | --- | --- | --- | --- | --- |
| After Treatment | 1.693 | 1.268 | | 1.340 | 0.188 | | | -0.855 | | | 4.241 | | |  | | |
| After Treatment × Nudge | -1.267 | 0.934 | | -1.360 | 0.181 | | | -3.143 | | | 0.610 | | |  | | |
| After Treatment × Boost | -1.556 | 1.062 | | -1.460 | 0.149 | | | -3.691 | | | 0.579 | | |  | | |
| Constant | 20.202 | 0.261 | | 77.390 | 0.000 | | | 19.678 | | | 20.727 | | | *** | | |
| Within R-squared | 0.068 | | Number of obs. (users) | | | 3876 (140) | | |  | | |  | | |  |  |
| F-test | 1587816.52 | | Prob > F | | | 0.000 | | |  | | |  | | |  |  |
| Week fixed effects | Yes | | User fixed effects | | | | Yes | | |  | | |  | | |  |

Notes: Coefficients, standard errors and 95% confidence intervals from fixed-effects estimation with Driscoll-Kraay standard errors. The dependent variable is electricity consumption in kWh. Nudge and Boost are dummy variables which are 1 if the user was in the respective treatment group. After Treatment is a dummy equal to 1 after the treatment started for the respective participant. The dummies Nudge and Boost are dropped from the fixed effects estimation as they exhibit no time variation. Estimation includes weekly dummies to control for time trends that are not reported in the Table. *** p<.01, ** p<.05, * p<.1

Table S8: Pooled OLS regression results reported in Fig.6

| Competence | Coef. | | St.Err. | t-value | | p-value | [95% Conf | | | Interval] | | | Sig |  |  |
| --- | --- | --- | --- | --- | --- | --- | --- | --- | --- | --- | --- | --- | --- | --- | --- |
| After Treatment | -.353 | | .913 | -0.39 | | .699 | -2.155 | | | 1.448 | | |  |  |  |
| Nudge | -1.409 | | .910 | -1.55 | | .123 | -3.204 | | | .387 | | |  |  |  |
| Boost | -.535 | | .930 | -0.58 | | .566 | -2.37 | | | 1.299 | | |  |  |  |
| After Treatment × Nudge | 1.06 | | 1.282 | 0.83 | | .409 | -1.47 | | | 3.59 | | |  |  |  |
| After Treatment × Boost | 1.498 | | 1.344 | 1.11 | | .266 | -1.153 | | | 4.15 | | |  |  |  |
| Time spent with dashboard | .000 | | .000 | 3.08 | | .002 | .000 | | | .001 | | | *** |  |  |
| Constant | 5.903 | | .615 | 9.60 | | .000 | 4.69 | | | 7.116 | | | *** |  |  |
| R-squared | | 0.050 | | Number of obs. | | | | 188 | | |  | | | |  |
| F-test | | 2.000 | | Prob > F | | | | 0.068 | | |  | | | |  |
| Akaike crit. (AIC) | | 1030.695 | | | Bayesian crit. (BIC) | | | | 1053.350 | | |  | | | |
| Notes: Coefficients, robust standard errors and 95% confidence intervals from a pooled OLS estimation. The dependent variable is a competence score from a competence test conducted before the treatment started and at the end of the experiment. Nudge and Boost are dummy variables which are 1 if the user was in the respective treatment group. After Treatment is a dummy variable equal to 1 after the treatment started for the respective participant. Time spent with dashboard indicates the total time that the participant engaged with the provided user dashboard. *** p<.01, ** p<.05, * p<.1 | | | | | | | | | | | | | |  |  |

Table S9: Fixed effects regression results reported in Fig.6

| Competence | Coef. | | Std. Err. | | t-value | p-value | | | [95% Conf. | | | Interval] | | | Sig. | | |
| --- | --- | --- | --- | --- | --- | --- | --- | --- | --- | --- | --- | --- | --- | --- | --- | --- | --- |
| After Treatment | -.071 | | .643 | | -0.11 | .912 | | | -1.345 | | | 1.202 | | |  | | |
| After Treatment × Nudge | 1.113 | | 1.027 | | 1.08 | .281 | | | -.922 | | | 3.148 | | |  | | |
| After Treatment × Boost | 1.976 | | .992 | | 1.99 | .049 | | | .011 | | | 3.942 | | | ** | | |
| Constant | 5.467 | | .207 | | 26.43 | 0 | | | 5.057 | | | 5.876 | | | *** | | |
| Within R-squared | | 0.100 | | Number of obs. (part.) | | | 188 (115) | | |  | | |  | | |  |  |
| F-test | | 2.683 | | Prob > F | | | 0.073 | | |  | | |  | | |  |  |
| Week fixed effects | | No | | User fixed effects | | | | Yes | | |  | | |  | | |  |

Notes: Coefficients, clustered standard errors and 95% confidence intervals from fixed-effects estimation. The dependent variable is a competence score from a competence test conducted before the treatment started and at the end of the experiment. Nudge and Boost are dummy variables which are 1 if the user was in the respective treatment group. After Treatment is a dummy variable equal to 1 after the treatment started for the respective participant. The dummies Nudge and Boost as well as the count of time spent with dashboard are dropped from the fixed effects estimation as they exhibit no time variation. *** p<.01, ** p<.05, * p<.1

Table S10: Fixed effects regression results in the double-moderator analysis reported in Fig.7

| Consumption | Coef. | Std. Err. | t-value | p-value | [95% Conf. | Interval] | Sig. |
| --- | --- | --- | --- | --- | --- | --- | --- |
| **High Competences and High Environmental Motivation** (N = 70, n = 3059) | | | | | | |  |
| After Treatment | 8.613 | 1.360 | 6.330 | 0.000 | 5.883 | 11.344 | *** |
| After Treatment × Nudge | -4.314 | 1.462 | -2.950 | 0.005 | -7.250 | -1.378 | *** |
| After Treatment × Boost | -3.270 | 1.053 | -3.110 | 0.003 | -5.383 | -1.157 | *** |
| Constant | 21.289 | 0.374 | 56.920 | 0.000 | 20.538 | 22.040 | *** |
| **Low Competences and High Environmental Motivation** (N = 18, n = 814) | | | | | | | |
| After Treatment | 2.541 | 2.253 | 1.13 | .275 | -2.213 | 7.295 |  |
| After Treatment × Nudge | .18 | 1.451 | 0.12 | .902 | -2.881 | 3.242 |  |
| After Treatment × Boost | .479 | 1.031 | 0.46 | .648 | -1.697 | 2.654 |  |
| Constant | 17.283 | 1.331 | 12.98 | 0 | 14.474 | 20.092 | *** |
| **High Competences and Low Environmental Motivation** (N = 27, n = 1256) | | | | | | | |
| After Treatment | 1.832 | 1.328 | 1.380 | 0.174 | -0.834 | 4.497 |  |
| After Treatment × Nudge | 0.958 | 1.933 | 0.500 | 0.622 | -2.924 | 4.839 |  |
| After Treatment × Boost | -1.040 | 0.915 | -1.140 | 0.261 | -2.876 | 0.797 |  |
| Constant | 21.957 | 0.151 | 145.630 | 0.000 | 21.654 | 22.260 | *** |
| **Low Competences and Low Environmental Motivation** (N = 25, n = 1163) | | | | | | | |
| After Treatment | 1.832 | 1.328 | 1.380 | 0.174 | -0.834 | 4.497 |  |
| After Treatment × Nudge | 0.958 | 1.933 | 0.500 | 0.622 | -2.924 | 4.839 |  |
| After Treatment × Boost | -1.040 | 0.915 | -1.140 | 0.261 | -2.876 | 0.797 |  |
| Constant | 21.957 | 0.151 | 145.630 | 0.000 | 21.654 | 22.260 | *** |

Notes: Coefficients, Driscoll-Kraay standard errors and 95% confidence intervals from fixed-effects estimation. For the second group (low competences and high environmental motivation) clustered standard errors instead of Driscoll-Kraay standard errors are reported, as the latter could not be derived with N = 18. The dependent variable is a energy consumption in kWh. Nudge and Boost are dummy variables which are 1 if the user was in the respective treatment group. After Treatment is a dummy variable equal to 1 after the treatment started for the respective participant. The dummies Nudge and Boost are dropped from the fixed effects estimation as they exhibit no time variation. *** p<.01, ** p<.05, * p<.1

Table S11: Overview of hypotheses and adjusted p-values

| **Pre-registered Hypotheses** | | **Coef.** | **unadjusted** | **BH (FDR)** |
| --- | --- | --- | --- | --- |
|  | |  | **p-values** | |
| H1 | Boost vs. Control | -1.862 | 0.026 | 0.043 |
|  | Nudge vs. Control | -2.024 | 0.015 | 0.037 |
|  | Boost vs. Nudge | ***** |  |  |
| H2 | Boost vs. Control | 1.976 | 0.049 | 0.061 |
|  | Nudge vs. Control | 1.113 | 0.281 | 0.234 |
|  | Boost vs. Nudge | ***** |  |  |
| H3 | Boost vs. Nudge 1 | 0.024 | 0.974 | 0.487 |
|  | Boost vs. Nudge 2 | 1.217 | 0.216 | 0.216 |
|  | Boost vs. Nudge 3 | -0.29 | 0.578 | 0.321 |
| H4 | Boost vs. Control 1 | -2.61 | 0.016 | 0.037 |
|  | Boost vs. Control 2 | -2.869 | 0.002 | 0.020 |
|  | Boost vs. Control 3 | -1.556 | 0.149 | 0.149 |

Notes: We applied the Benjamini-Hochberg procedure to control the false discovery rate (FDR) at 5%. *** means that the hypothesis was not formally tested as coefficients are close and CIs widely overlap.

**Figures**

Figure S1: Mean self-efficacy (upper panel) and norm salience (lower panel) scores

Notes: Bar graphs show mean self-efficacy and norm-salience scores and 95% confidence intervals in the treatment and control groups. Measures were elicited before the treatment started and after the experiment concluded.

Figure S2: Estimation results for energy consumption in 6-week intervals

Notes: The blue data points show coefficients and 95% confidence intervals for weeks 0 to 5, the red for weeks 6 to 11, and the green for weeks 12 to 17. The “After Treatment” dummy represents the Control group. Estimation results are from fixed-effects estimation with Driscoll-Kraay standard errors. All estimations include weekly dummies to control for time trends that are not reported in the graph.

Figure S3: Treatment engagement in the Nudge and Boost condition

(A) Weekly average tip use in the Boost treatment

(B) Average weekly dashboard usage in the Nudge Treatment

Figure S4: Estimation results of double-moderator analysis

Notes: The graphs show coefficients and 95% confidence intervals of the interaction terms from fixed-effects estimations with Driscoll-Kraay standard errors. Only the red group (low competences and high motivation) was estimated with fixed effects estimation using clustered standard errors as Driscoll-Kraay standard errors could not be derived for this group of N = 18 participants. The dummies Nudge and Boost are dropped from the estimation, After Treatment is not reported in the graphs. All estimations include weekly dummies to control for time trends that are not reported in the graph.

Figure S5: An illustrative representation of the participant’s dashboard in the Nudge treatment

Figure S6: A visual representation of the QR code stickers used in the Boost treatment
